# Peripheral Neuropathy in Parkinson’s Disease Treated with Device-Assisted Therapies: How Often It Is Reported Depends on How It Is Sought. A Systematic Review and Meta-Analysis

**DOI:** 10.3390/biomedicines14102279

**Published:** 2026-10-08

**Authors:** Natalia Blidaru, Delia Tulbă, Teodor Cristian Blidaru, Alin Marian Stoleru, Ana-Maria Constantinescu, Mădălina Elena Popescu, Dragos Nicolae Garofil, Bogdan Ovidiu Popescu

**Affiliations:** 1 Faculty of Medicine, “Carol Davila” University of Medicine and Pharmacy, 37 Dionisie Lupu Street, 020021 Bucharest, Romania; natalia-ioana.dima@drd.umfcd.ro (N.B.); delia.tulba@umfcd.ro (D.T.);; 2 Department of Neurology, Colentina Clinical Hospital, 19-21 Ștefan cel Mare Road, 020125 Bucharest, Romania; 3 Colentina Research and Development Center, Colentina Clinical Hospital, 19-21 Ștefan cel Mare Road, 020125 Bucharest, Romania; 4 Laboratory of Cell Biology, Neurosciences and Experimental Myology, “Victor Babeș” National Institute of Pathology, 99-101 Splaiul Independenței, 050096 Bucharest, Romania

**Keywords:** Parkinson’s disease, polyneuropathy, device-aided therapy, levodopa–carbidopa intestinal gel, catechol-O-methyltransferase inhibition, entacapone, homocysteine, systematic review, meta-analysis

## Abstract

**Background/Objectives**: Peripheral neuropathy (PN) is an increasingly recognized complication of device-assisted therapies for advanced Parkinson’s disease, with reported frequencies ranging from 1% to nearly 50% and no pooled estimate available. We aimed to estimate how often PN is reported under device-assisted therapy and whether its frequency is associated with levodopa exposure, catechol-O-methyltransferase (COMT) inhibition, and vitamin status. **Methods:** PubMed, Scopus, and Web of Science were searched without date restriction, and the reference lists of 21 reviews were screened. Studies reporting a peripheral nerve outcome under any device-assisted therapy were eligible, and proportions were pooled using random-effects models (PROSPERO CRD 1375888). **Results:** Seventy-five studies with 7106 patients were included. The pooled frequency under levodopa–carbidopa intestinal gel (LCIG) was 13.2% (95% confidence interval 9.8 to 17.6), against one case among 272 patients under levodopa-entacapone-carbidopa intestinal gel (LECIG). Frequency rose with levodopa dose (odds ratio 1.28 per 100 mg/day) and treatment duration (1.30 per 12 months). Neither COMT inhibition nor prophylactic B-vitamin supplementation was associated with lower frequency. Reported frequency ranged from 3.9%, where PN was recorded only when volunteered, to 33.6%, where all patients were tested. **Conclusions:** How intensively PN is sought appears to be a major determinant of how often it is found.

## 1. Introduction

### 1.1. Background

Parkinson’s disease is the second-most common neurodegenerative disorder. It is characterized pathologically by the loss of dopaminergic neurons in the substantia nigra and by intraneuronal α-synuclein aggregation, and clinically by bradykinesia with rigidity or rest tremor, accompanied by a broad range of non-motor manifestations. The combination of levodopa and a dopa decarboxylase inhibitor remains the most effective symptomatic treatment, but when motor complications are no longer adequately controlled with optimized oral therapy, device-assisted therapies may be considered in appropriately selected patients [1]. These comprise deep brain stimulation, most often of the subthalamic nucleus; the subcutaneous infusions of apomorphine and foslevodopa–foscarbidopa; and the intrajejunal gels levodopa–carbidopa (LCIG) and levodopa–entacapone–carbidopa (LECIG). Subcutaneous levodopa–carbidopa (ND0612) is in clinical development.

Peripheral neuropathy (PN) has emerged as a clinically relevant but often under-recognized problem in Parkinson’s disease, adding to the considerable non-motor burden of the disorder. It may present with distal sensory loss, paresthesia, neuropathic pain, weakness, or gait impairment, while milder abnormalities may remain subclinical and become apparent only with systematic testing. Toth and colleagues [2] were among the first to highlight the unexpectedly high frequency of PN in Parkinson’s disease, prompting increasing interest in whether peripheral nerve involvement reflects the disease process itself, treatment-related factors, or both. The metabolic argument for the latter runs as follows. Methylation of levodopa by catechol-O-methyltransferase (COMT) consumes S-adenosylmethionine and generates S-adenosylhomocysteine, which is hydrolyzed to homocysteine; sustained levodopa exposure therefore raises plasma homocysteine. Vitamins B6, B12 and folate are the cofactors of homocysteine metabolism, B12 and folate supporting its remethylation to methionine and B6 its transsulfuration to cysteine. A persistently increased homocysteine load therefore draws on these vitamins, and their functional sufficiency may fall over time [3,4]. Vitamin B6 is additionally depleted by a route that does not involve methylation at all. It is the cofactor of the dopa decarboxylase reaction, and carbidopa binds it irreversibly, so the decarboxylase inhibitor given alongside levodopa inactivates the vitamin whether or not the methylation pathway is engaged [5,6]. Methylmalonic acid rises mainly as a marker of functional B12 deficiency rather than as an independent product of this pathway. If this sequence contributes to nerve injury, inhibiting COMT may be protective, since less levodopa would be diverted toward methylation. Two studies in orally treated patients are consistent with that hypothesis: Cossu and colleagues reported a lower risk of PN in patients taking a COMT inhibitor [7], and Grofik and colleagues reached a similar conclusion for entacapone [8]. The argument has been extended to LECIG, in which entacapone is delivered within the gel itself [9].

Enteral levodopa warrants particular attention, as it produces higher and steadier levodopa exposure than intermittent oral dosing. Reported frequencies of PN under enteral levodopa nevertheless range from 1% to nearly 50%. Antonini and colleagues report 1.1% from the serious adverse-event code of the GLORIA registry [10], and Romagnolo and colleagues report 42.1% [11]. Piekarski and colleagues note that studies designed to detect PN report between 13.8% and 100%, whereas large multicenter safety studies report between 4.5% and 13% [12]. Previous reviews have attributed this spread mainly to differences in how PN was sought [11,12,13,14], without quantifying it.

To our knowledge no meta-analysis of this question has been published, so previously reported figures (16.3% and 56.9% [13], 30.2% and 42.1% [11], and 41.4% [14]) are summed proportions reported without weights or confidence intervals. These estimates continue to be cited in recent reviews. A 2025 systematic review of PN in Parkinson’s disease reproduces two of them unchanged [15], and a 2026 review states that neuropathy is present in approximately 42% of patients receiving LCIG [6]. A second gap is that prevalent and incident PN have rarely been distinguished, which limits any assessment of treatment-emergent neuropathy [1,11]. Finally, we are not aware of a systematic search restricted to device-assisted therapy conducted since early 2023. The reviews published since address peripheral neuropathy in Parkinson’s disease as a whole [15,16] or across parkinsonian syndromes [6], which leaves LECIG, foslevodopa–foscarbidopa and ND0612 outside the existing syntheses at a time when their use is expanding.

### 1.2. Objectives

We aimed to estimate the reported frequency of PN in patients receiving device-assisted therapy and to describe its distribution across device modalities. We further sought to assess whether the reported frequency is associated with levodopa exposure, COMT inhibition, biochemical alterations and B-vitamin supplementation. We also aimed to describe the clinical course of PN, and the management measures taken in response to it, and to quantify the influence of PN ascertainment methods on the frequencies reported. We hypothesized that the reported frequency of PN would be associated with greater levodopa exposure and would be lower in cohorts receiving COMT inhibition. These hypotheses were specified in the registered protocol; all other analyses reported here are exploratory.

## 2. Materials and Methods

### 2.1. Protocol

The protocol was registered with PROSPERO before data extraction began (CRD 1375888), and the review is reported according to the PRISMA 2020 checklist [17], provided as Table S1. The review addressed the following question: “In patients with Parkinson’s disease receiving device-assisted therapy, what is the reported frequency of peripheral neuropathy, and is that frequency associated with levodopa exposure, COMT inhibition and vitamin status?”

### 2.2. Eligibility Criteria

The target population consisted of adult patients with Parkinson’s disease receiving device-assisted therapy, comprising LCIG, LECIG, continuous subcutaneous foslevodopa–foscarbidopa, subcutaneous levodopa–carbidopa (ND0612), continuous subcutaneous or intravenous apomorphine, and deep brain stimulation of the subthalamic nucleus or globus pallidus internus. The outcome was PN diagnosed clinically, electrophysiologically by nerve conduction studies (NCS) or electromyography, morphometrically by intraepidermal nerve fiber density, or by any other validated method of peripheral nerve assessment. Randomized trials, prospective and retrospective cohorts, cross-sectional studies and case series were eligible; no language restriction was applied.

Four further rules were applied.

First, case series had to report more than three patients. A proportion derived from one, two, or three patients carries no useful precision and would enter a pooled estimate with a weight disproportionate to its information content, and reports of that size are selected for publication because they are unusual. Eight reports fall below the threshold. They include Urban and colleagues, who described two patients with subacute axonal PN under LCIG and combined vitamin B12 and B6 deficiency [18], and Galazky and colleagues, whose two patients recovered only after device withdrawal [19].

Second, only primary data were eligible; narrative reviews were excluded even where they addressed the question closely [20].

Third, the outcome had to be a generalized polyneuropathy. Focal and iatrogenic mononeuropathies were excluded as a distinct entity with a different mechanism, which removes Loizon and colleagues, reporting carpal tunnel syndrome in 9/115 stimulated patients [21].

Fourth, acute immune-mediated polyradiculoneuropathies were excluded, since their pathophysiology and time course differ from the distal axonal neuropathy this review addresses and their occurrence after a surgical procedure raises a separate causal question. This removes Theuriet and colleagues, reporting Guillain-Barré syndrome twelve days after subthalamic nucleus implantation [22]. Cases of acute or subacute demyelinating neuropathy arising during infusion therapy, which were reported within otherwise eligible cohorts, were retained and are described in Section 3.6.

Studies reporting only biochemical abnormalities, without clinical, electrophysiological or morphometric assessment, did not address the primary outcome and were not eligible. They are cited in the Discussion where relevant to the mechanistic argument but form no part of the synthesis, in keeping with previous reviews in this field [11,12,13,14].

### 2.3. Search and Study Selection

PubMed, Scopus and Web of Science were searched from the earliest record available in each database to 22 April 2026, and the reference lists of 21 relevant reviews were screened manually through 15 August 2026. The full search strategy and the execution date of each component are provided in Table S2. Titles and abstracts, and then full texts, were screened independently by two reviewers. Any disagreement was to be resolved by discussion, with a third reviewer available for arbitration; arbitration was not needed. Included studies were checked for duplicate publication and for cohort overlap; eleven overlap clusters were identified, and 16 studies were excluded from the pooled analyses because another report of the same patients was used instead (Table S3).

### 2.4. Data Extraction

Thirteen studies report two or more treatment arms, so data were extracted per arm and per estimate rather than one row per study, with laboratory values and individually described patients held in separate linked tables. For each estimate, we recorded the numerator, the denominator and the population it describes, the case definition in the authors’ own words, and the instruments used. Percentages printed by the authors were recorded but recalculated from the counts.

The denominator throughout is the number of patients actually assessed for PN, which is often smaller than the number enrolled. In 22 studies, the figure quoted in the abstract is not the one the neuropathy percentage was calculated on; in the GLORIA registry, for example, the reported 4.5% refers to the safety population rather than to the full enrolled cohort.

A second reviewer re-read the sources and adjudicated the classifications, which produced nineteen corrections (Table S4).

We also recorded the PN ascertainment strategy of each estimate, taken from the methods section of each paper. Five strategies occur: PN recorded only when reported spontaneously as an adverse event; examination or NCS only in symptomatic patients; examination of all patients without neurophysiology; NCS in all patients; and NCS with small-fiber assessment in all patients. The classification rests on who was assessed rather than on the instrument alone, so a cohort tested by NCS only after symptoms appeared belongs to the second strategy and not the fourth.

### 2.5. Risk of Bias Assessment

Randomized trials were appraised with RoB 2 [23] and non-randomized studies with a comparator using ROBINS-I [24]. Single-arm prevalence estimates were appraised with the Joanna Briggs Institute (JBI) checklist for prevalence studies [25] and case series with the JBI case-series checklist. Judgments were made for each domain and are given in Table S5. Appraisal was carried out at the level of the estimate rather than the study. A study contributing both a single-arm proportion and a comparison was therefore appraised with more than one instrument, and the appraisal counts exceed the number of studies.

### 2.6. Synthesis

Proportions were analyzed on the logit scale. In cases where an arm reported no events, or events in every patient, a continuity correction of 0.5 was added to the numerator and 1.0 to the denominator; all other arms were used as reported. Estimates were combined by a random-effects model with the DerSimonian–Laird estimator of between-study variance [26] and inverse-variance weighting, and pooled values were returned to the percentage scale by the inverse logit. Heterogeneity is reported as Cochran’s Q and I^2^ [27], and subgroups were compared by the between-group Q test. All eligible estimates were included; an arm with a single patient yields no proportion and was excluded. Groups with fewer than two estimates, and groups in which only one event was observed, are reported as counts with a Wilson interval rather than pooled. The same applies to a group of two estimates at opposite extremes of the scale, with no events in one arm and events in every patient in the other. Both estimates then carry a continuity correction, and a pooled value would reflect the correction rather than the data. This rule was not pre-specified. It was applied to one group, subcutaneous apomorphine (0/39 and 4/4), which Table 1 reports as counts.

The primary summary measure, referred to throughout as the reported frequency of PN, combines point prevalence, period prevalence and adverse-event frequency, because the ascertainment method determines which of them a study is able to report. Cumulative incidence was analyzed separately.

Associations with study-level covariates were examined by univariable random-effects meta-regression on the logit scale, with the between-study variance estimated for each model from its own residual Q. These models are exploratory and were not adjusted for multiplicity. Whether COMT inhibition modifies the association between levodopa exposure and PN was considered but not estimated, since only 11 estimates report both a levodopa-equivalent dose and the proportion of patients taking a COMT inhibitor. Biomarker concentrations reported separately for patients with and without PN in the same cohort were combined as mean differences on the natural scale of each analyte. Where a study reported a median with quartiles, the mean and standard deviation were derived from them by the method of Wan and colleagues [28].

Prophylactic supplementation from treatment initiation was compared with no supplementation. Deficiency-triggered supplementation and unreported policies were analyzed separately, since neither is a test of prevention. Because supplementation policy proved to be associated with the intensity of the work-up, the comparison was repeated among cohorts in which every patient underwent nerve conduction studies. In a post hoc sensitivity analysis added at peer review, it was also repeated among estimates of cumulative incidence, whose denominators exclude patients with PN before device initiation.

Three sensitivity analyses were pre-specified: an alternative moment estimator of the between-study variance [29], a continuity correction of 0.1 instead of 0.5, and restriction to arms with at least five patients.

All analyses were performed in Microsoft Excel for Microsoft 365 (Microsoft Corporation, Redmond, WA, USA). The full workbook, with live formulas from the extracted counts through to every result reported here, is provided as File S1, together with the implementation details of the meta-regression and the variance estimators.

## 3. Results

### 3.1. Study Selection and Characteristics

**Figure 1:**
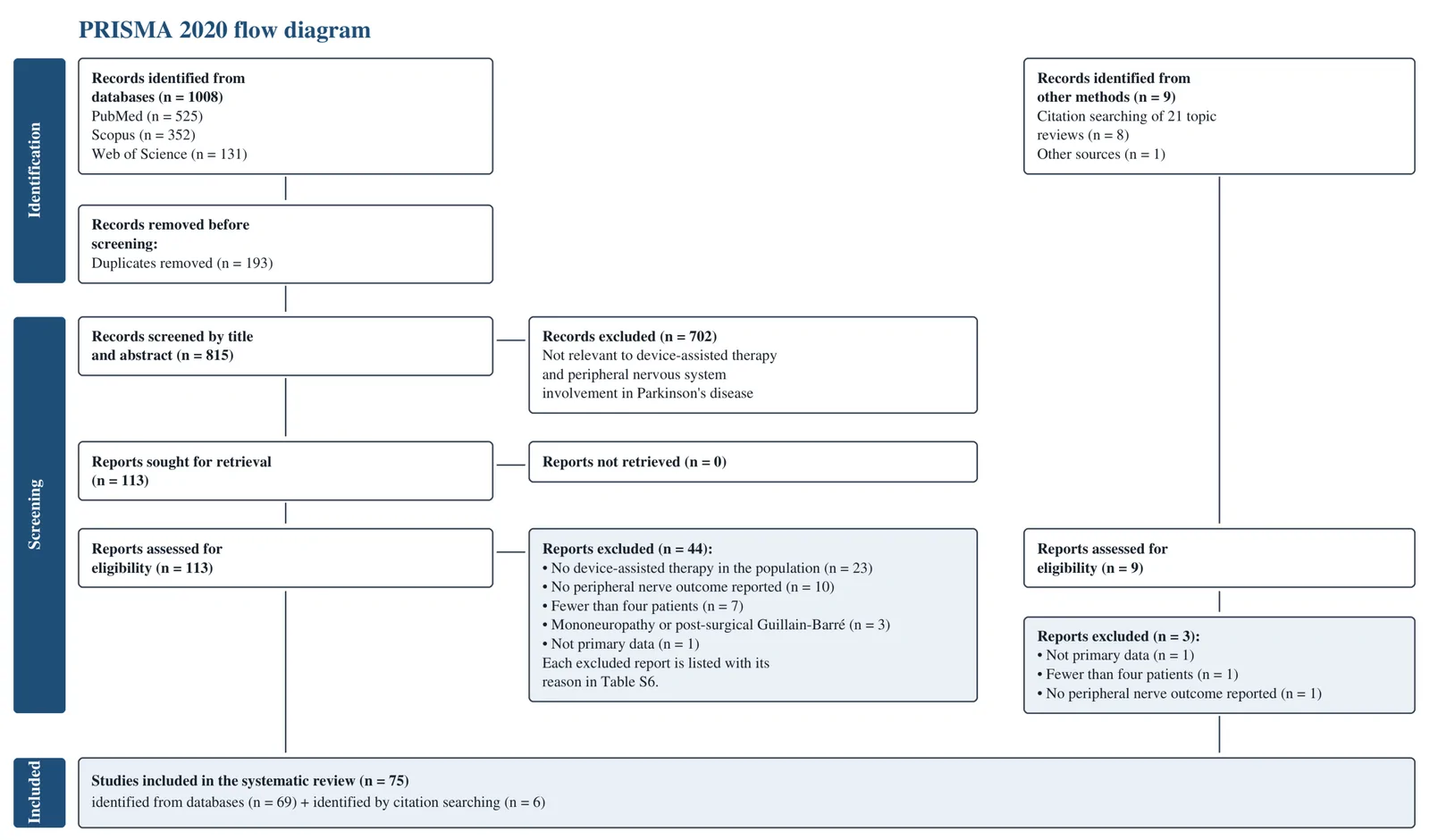
PRISMA 2020 flow diagram of study selection for this review of peripheral neuropathy in Parkinson’s disease treated with device-assisted therapies (PROSPERO CRD 1375888). Reports excluded after full-text assessment are grouped by reason; each is listed individually in Table S6. Of the 75 included studies, 73 report a polyneuropathy case count; the remaining two assessed it systematically, clinically and electrophysiologically, but reported it only as a continuous outcome.

The search identified 1008 records. After removal of 193 duplicates, 815 were screened by title and abstract and 702 were excluded. All 113 remaining full texts were assessed and 44 were excluded, most often because the cohort had received no device-assisted therapy (*n* = 23) or because no peripheral nerve outcome was reported (*n* = 10). The reasons are grouped in Figure 1, and each excluded report is listed individually in Table S6. Citation searching of the reference lists of 21 topic reviews identified eight further reports and one more was identified from another source; six of these nine were included. Seventy-five studies were included in the review (Figure 1) [10,30,31,32,33,34,35,36,37,38,39,40,41,42,43,44,45,46,47,48,49,50,51,52,53,54,55,56,57,58,59,60,61,62,63,64,65,66,67,68,69,70,71,72,73,74,75,76,77,78,79,80,81,82,83,84,85,86,87,88,89,90,91,92,93,94,95,96,97,98,99,100,101,102,103].

The two studies without a case count (Figure 1 legend) contribute to the biochemical and neurophysiological sections instead. The 75 studies were published between 2007 and 2026 and comprise 117 arms, 91 of which received a device, and 176 outcome estimates, with 7106 patients assessed for PN. Withholding the 16 reports whose patients overlap with another included report (Table S3) leaves 5022 distinct patients.

Designs were predominantly observational: 22 prospective and 20 retrospective single-arm cohorts, eight analytical cross-sectional studies, seven non-randomized comparative cohorts, five registry cohorts, four case series and four randomized trials, alongside two pooled analyses of trial programs and three further reports. Southern Europe contributed 32 studies, Western Europe 11, Central and Eastern Europe 10, North America six, Northern Europe four and the Middle East and North Africa four, with one from Oceania; seven were multinational. LCIG dominated, accounting for 76/91 device arms, while LECIG contributed five arms, subthalamic deep brain stimulation two, apomorphine two, foslevodopa–foscarbidopa two, ND0612 two, and another stimulation target or a combination of devices two.

Across arms, median age was 66.6 years (interquartile range 64.7–68.4), median disease duration 13.0 years (11.9–14.2), median Hoehn and Yahr stage 3.0, and median levodopa-equivalent daily dose 1319 mg (1164–1516). Median device exposure was 24 months, ranging from under three months to more than fourteen years, with infusion delivered for a median of 16 h/day. Fifty-one studies declared an industry relationship among the authors, 19 did not, and five were unclear. Study-by-study characteristics are detailed in Table S7.

### 3.2. Frequency of Peripheral Neuropathy by Device Modality

Pooled frequencies differed between modalities (Table 1; Q = 13.9, df = 2, *p* = 0.001). The comparison, however, is confounded by differences in ascertainment methods and in the amount of available evidence, and should not be read as a ranking of risk.

LCIG has by far the largest evidence base, with 42 contributing estimates, although heterogeneity remains substantial (I^2^ = 85%). Six LECIG cohorts were included, four of which contributed to the frequency estimate; three of these reported no PN cases. Because the data are so sparse, the pooled estimate changes noticeably depending on the continuity correction applied to zero-event studies, particularly because one cohort included only five patients. We therefore considered the aggregate proportion of 1/272 (0.4%) more informative than a pooled estimate. Of the two that did not contribute, one is a comparator arm of 14 patients in an LCIG study, with no cases, and one is discussed in Section 3.3. The subthalamic deep brain stimulation figure rests on two arms of five and 101 patients, and its interval spans almost the entire possible range. Foslevodopa–foscarbidopa and ND0612 each rest on a single trial, and the two apomorphine arms, of 39 and four patients, report 0% and 100%; all three are given as counts.

Twenty-four estimates restricted their denominator to patients known to be free of PN before device initiation. Pooled cumulative incidence of new-onset PN was 13.7% (95% CI 9.4 to 19.5; I^2^ = 63%) across 789 patients at risk. Baseline nerve status is reported in only a minority of studies, so those able to supply an incidence estimate represent a subset of studies with documented baseline PN status.

### 3.3. Levodopa Exposure and COMT Inhibition

**Figure 2:**
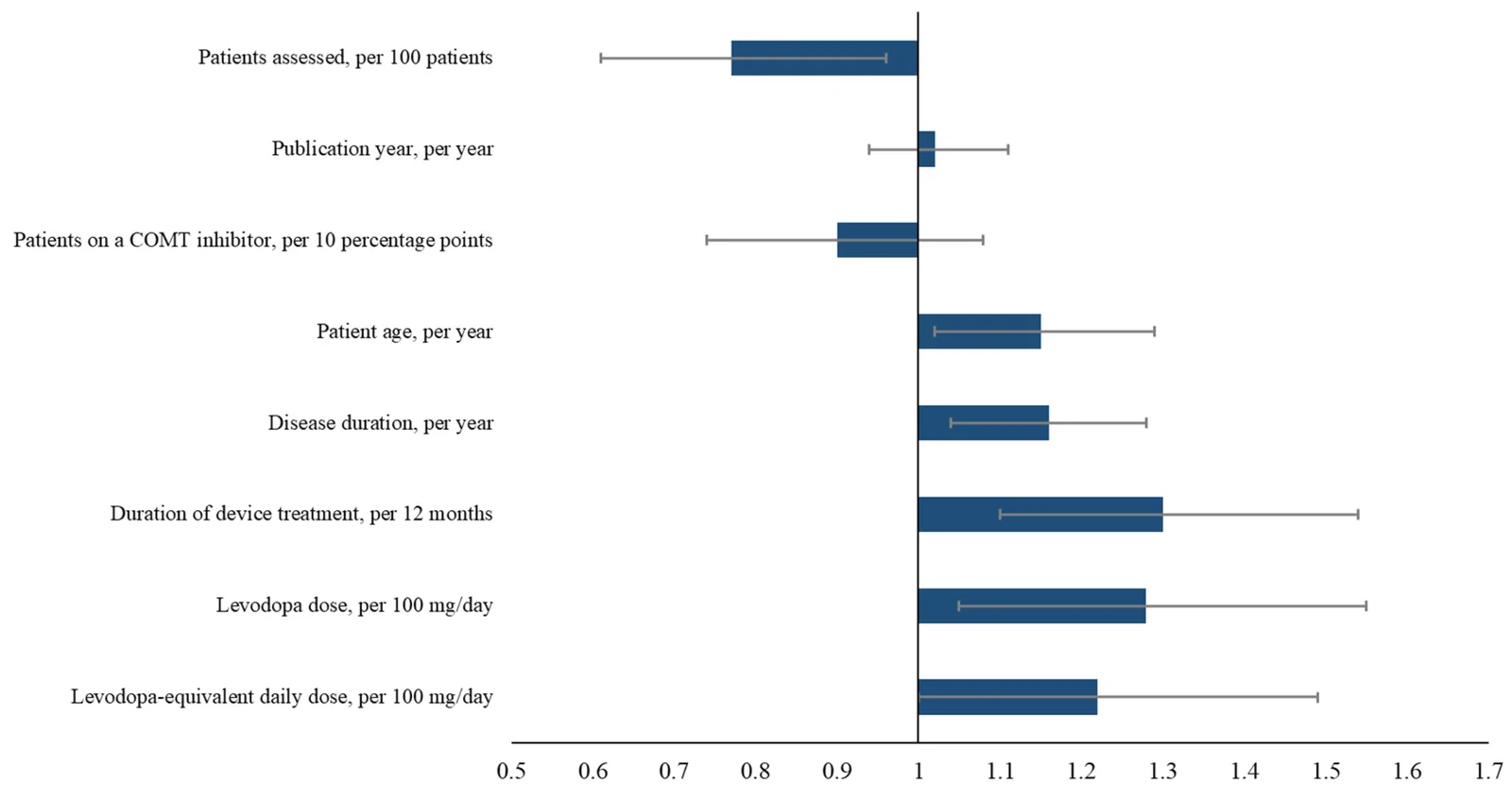
Univariable random-effects meta-regression of the reported frequency of peripheral neuropathy on study-level covariates. Bars are the odds ratios given in Table 2, drawn from the null value of 1, with 95% confidence intervals. The chart was produced in Microsoft Excel from the values in File S1.

PN was reported more frequently in cohorts receiving higher levodopa doses and undergoing longer device treatment (Table 2, Figure 2). The direction was the same for levodopa dose and for duration of device treatment, while the association with levodopa-equivalent daily dose was of similar magnitude but did not reach statistical significance. Disease duration and patient age were also associated with reported frequency. This finding is relevant to the interpretation of the results, as patients receiving higher doses for longer periods are likely to have more advanced disease, and treatment exposure cannot be separated from disease progression in these data. Reported frequency was lower in larger studies, a pattern consistent with small-study effects.

Several reports describe PN emerging during a period of dose escalation. In the only randomized comparison of LCIG with oral levodopa [72], oral levodopa in the comparator arm rose by 249.7 mg/day against 91.7 mg/day in the LCIG arm, and the arm with the greater escalation reported more PN symptoms.

Forty-three of the 91 device arms report how many patients were taking a COMT inhibitor, with a median of 29% per arm; of these, 21 contributed an estimate that entered the meta-regression, which found no significant association (Table 2). This is a study-level analysis rather than a patient-level comparison, and only two included studies directly compared COMT-treated and untreated patients within the same cohort. In the randomized one, opicapone was added to LCIG in 11 of 22 patients already on a B-vitamin supplement. Four of eleven in each arm had PN at randomization, and neither arm had a new or worsening case at twelve months [44]. In the other study, sixteen LCIG patients were assigned by eligibility to add tolcapone, to receive intramuscular B12 with folate, or to receive neither, and the polyneuropathy score fell only under tolcapone [96]. No included study reports PN counts cross-classified by COMT use, so an odds ratio quantifying the association between neuropathy and COMT inhibition cannot be assembled; the biochemical findings of these two studies are reported in Section 3.4.

LECIG delivers entacapone within the gel, thereby providing the closest approximation to a natural experiment; however, the comparison with LCIG remains indirect. Of six LECIG cohorts, four contribute an estimate: three report no cases, and the ELEGANCE registry reports one sensory neuropathy among 167 patients, first noted about three months after initiation [101], giving one case among 272 patients. A Romanian single-center cohort of 50 patients reports two further cases [73] but was excluded from every pooled analysis because its patients probably overlap the ELEGANCE registry and the two cases belong to a subset whose size is not stated. In a post hoc analysis entering those cases against the full cohort of 50, the aggregate moves from 1/272 to 3/322, which does not change the interpretation. The evidence is weaker than the counts suggest: of the 272 patients contributing, only five underwent nerve conduction studies.

### 3.4. Vitamin B Status, Homocysteine and Supplementation

Vitamin B12 was measured in 25 studies, homocysteine in 19, folate in 18 and vitamin B6 in 12; methylmalonic acid was measured in three and holotranscobalamin in one. Not every measurement is usable: a numeric value is given for vitamin B12 in 21 studies, homocysteine in 17, folate in 17 and vitamin B6 in 8, the remainder reporting only how many patients fell outside a reference limit. No included study reported a quantitative 3-O-methyldopa or homovanillic acid value. What each analyte can and cannot support is set out in Table S8.

Three questions are separable in these data, and are treated separately here: whether the biomarkers change under treatment, whether they differ between the patients who develop PN and those who do not, and whether giving vitamins prophylactically reduces PN.

#### 3.4.1. Change Under Treatment

Five cohorts report a whole-cohort homocysteine value both before the device was started and during treatment. It rose in all three LCIG cohorts, from 42.3 to 43.4, from 16.7 to 17.8 and from 15.7 to 28.8 µmol/L [68,76,94], and fell in both LECIG cohorts, from 18.0 to 15.0 and from 14.9 to 12.8 µmol/L [86,100]. The separation is complete and consistent with the direction predicted by the metabolic hypothesis, entacapone being delivered within the LECIG gel, but it rests on five cohorts that differ in many respects besides the formulation. Vitamin B12 levels showed a less consistent pattern: they fell in two LCIG cohorts [39,68], in one after a transient rise at one month, rose in one under supplementation [94], and were unchanged in both LECIG cohorts. Folate fell in the two LCIG cohorts reporting it longitudinally [39,68] and rose in the one LECIG cohort [100]. Vitamin B6 rose in the only two cohorts that gave a numeric value before and during treatment, both LECIG [86,100].

#### 3.4.2. Difference Between Patients with and Without PN

Three studies report vitamin B12, homocysteine and folate separately for patients with and without PN within the same cohort [55,65,80]. Vitamin B12 and folate were consistently lower in the patients with PN, with no detectable heterogeneity, while homocysteine was higher in two of the three and lower in the third, leaving the pooled difference imprecise (Table 3). The study that showed an inverse direction of association compared with patients with pre-existing PN and patients without it, before device initiation [80]. In the other two cohorts, homocysteine was 11.8 and 11.2 µmol/L higher in the patients with PN. Vitamin B6 was reported by PN status in only one study, at 16.3 against 19.5 ng/mL, a difference the authors report as non-significant [55]. No study reports how many patients were below a deficiency threshold separately for those with and without PN, so the more intuitive comparison, an odds ratio for PN given deficiency, cannot be assembled from the published data.

#### 3.4.3. Biomarkers Under COMT Inhibition

The two studies that compare COMT-treated and untreated patients within one cohort both measured the biochemistry, and they agree. Over six months, homocysteine rose by 4.95 µmol/L in the patients given nothing and fell by 8.81 under tolcapone and by 17.73 under B vitamins [96]. At twelve months it was 12.7 against 17.6 µmol/L in the opicapone and control arms (*p* = 0.053) [44]. Vitamin B12 moved reciprocally, falling by 32.6 pg/mL without treatment and rising by 32.7 under tolcapone [96], and reaching 348.9 against 247.1 pg/mL in the randomized comparison (*p* = 0.015), although both arms took the same supplement throughout [44]. Neither study was designed to detect a difference in neuropathy, and the tolcapone comparison is unrandomized and accompanied by a levodopa dose reduction.

#### 3.4.4. Prophylactic Supplementation

Eight studies gave B vitamins to every patient from the start of treatment, ten gave them only to patients found deficient, twenty gave none and thirty-seven did not report a policy. Only the first and third of these are comparable as a test of prevention. Deficiency-triggered supplementation is given after a deficiency has been identified, so the patients who receive it differ from those who do not in precisely the respect under study. The group whose policy is not reported describes the paper rather than the patients. Both are shown in Table 4 for completeness, but neither enters the comparison.

Prophylactic supplementation was not associated with a lower reported frequency than no supplementation (26.4% against 22.7%, Q = 0.14, df = 1, *p* = 0.71). That comparison is confounded by differences in the intensity of PN surveillance between the groups. Of the eight prophylactic estimates, four came from cohorts in which every patient underwent nerve conduction studies, against two of the eleven estimates from cohorts giving no supplementation. Restricting the comparison to cohorts in which every patient was tested did not change the result: 47.5% (32/67, k = 4) under prophylaxis against 70.4% (14/23, k = 2) without (Q = 0.52, *p* = 0.47). The point estimates ran the other way, but the group without supplementation contributed only two estimates. A further restriction to LCIG gave the same result (File S1).

Reported frequency includes patients whose PN predated the device, so the comparison was repeated in a post hoc sensitivity analysis restricted to new-onset PN. Among patients free of PN before device initiation, new-onset PN occurred in 12.0% (95% CI 3.1 to 36.4; 12/105, k = 6) under prophylactic supplementation. Without supplementation, it occurred in 13.6% (95% CI 6.5 to 26.4; 23/246, k = 6), a difference that was not significant (Q = 0.03, df = 1, *p* = 0.86; File S1). The intervals are wide, and surveillance again differed. Of the six prophylactic estimates, three came from cohorts in which every patient underwent nerve conduction studies and a fourth from the cohort that added small-fiber testing, against one of the six without supplementation.

The case reports point the same way: eight of the 57 individually described patients developed PN while already receiving prophylactic supplementation. In the one randomized comparison of COMT inhibition, a COMT inhibitor added to a universally supplemented LCIG cohort neither prevented nor modified PN, although vitamin B12 rose in the treated arm [44].

### 3.5. Large-Fiber and Small-Fiber Involvement

Almost all neurophysiological information in the included studies concerns large fibers: of the estimates that classify fiber type, 73 describe large-fiber involvement, three small fibers and three mixed. Four studies assessed small fibers and only one did so morphometrically in every patient. That study [48] enrolled five patients before LCIG initiation, repeating clinical examination, quantitative sensory testing, NCS and skin biopsy at 3, 6 and 12 months. One patient had mild epidermal denervation at baseline; by three months, all five showed epidermal denervation and raised thermal thresholds. Following one withdrawal, all four remaining patients developed burning pain with raised warm thresholds and severe skin denervation by six to twelve months, while only 1/4 had an abnormal NCS at twelve months. Two comparator groups of six patients each, on oral levodopa and levodopa-naïve, showed no such change.

No other included study assessed small-fiber function by skin biopsy, quantitative sensory testing, corneal confocal microscopy, or sudomotor testing. The implications of the discrepancy between the morphometric and the electrophysiological findings are considered in the Discussion.

### 3.6. Clinical Course and Management

Fifty-seven individually described patients were reported across 24 studies. Where the underlying pathology was classified, it was axonal in 35 and demyelinating in four, with four mixed; where modality was classified, 32 were sensorimotor and 15 purely sensory. Nerve involvement was described as severe in 20 patients and as disabling in 3.

Median latency from device initiation to PN onset was 6 months (interquartile range 4–12), ranging from under one month to four and a half years. Half of the described cases appeared within six months and three-quarters within one year. Twelve cases evolved acutely, over less than four weeks, twelve subacutely and eight followed a chronic course; in eight the PN was pre-existing and worsened, and seventeen could not be classified. Acute and subacute presentations therefore accounted for 24/40 classifiable cases. Individually described patients are a selected subset, and this proportion is not an estimate of the distribution of onset patterns among all patients who develop PN.

At cohort level, body mass index is reported in 20/91 device arms and weight in 17, and no study reports either separately for patients with and without PN, so no association can be quantified. Among individually described patients, 11/57 have weight loss, low body mass index, cachexia, or malnutrition recorded alongside the PN, across seven studies. These are two different levels of observation, and co-occurrence in case descriptions does not establish an association.

The device was withdrawn or interrupted in 27/57 described patients: 11/12 with an acute course, 8/12 subacute, 0/8 chronic, 4/8 in whom pre-existing PN worsened and 4/17 whose course could not be classified. An outcome was documented for 46 of the 57 patients; no course is reported for the remaining 11. Among those 46, 37 (80.4%) improved, stabilized, or resolved following vitamin supplementation, dose reduction or device withdrawal; three progressed despite intervention; five had the device discontinued specifically because of the PN and one was unchanged with no intervention given.

### 3.7. Influence of the Diagnostic Work-Up

**Figure 3:**
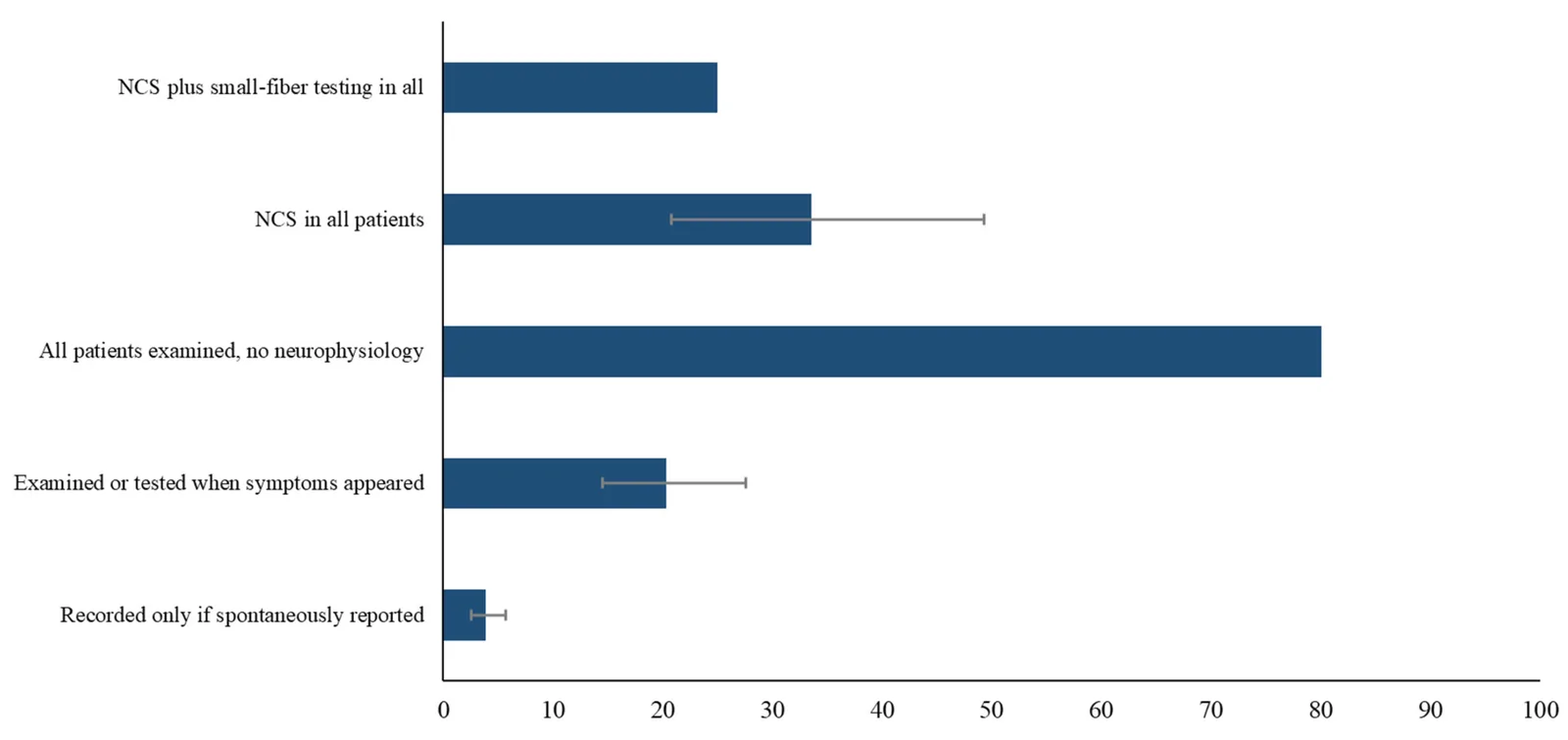
Reported frequency of peripheral neuropathy by PN ascertainment strategy, with 95% confidence intervals. The two strategies resting on 10 and four patients are shown without an interval, since they are reported descriptively rather than pooled. The chart was produced in Microsoft Excel from the values in File S1.

Of the factors examined, the PN ascertainment strategy showed the largest between-group difference (Table 5, Figure 3).

The difference between groups is significant (Q = 57.6, df = 2, *p* < 0.001), with an approximately nine-fold difference in reported frequency between studies recording PN only when spontaneously reported and studies testing every patient. Two groups are too small to pool. Only one study examined all patients without neurophysiological testing and reported a frequency in two arms comprising a total of 10 patients, and only one assessed small fibers in all patients, its denominator falling to four after one withdrawal. Pooling across all groups gives 11.2% (95% CI 8.3 to 15.1; I^2^ = 85%), a figure we report once for completeness, since it averages across groups defined by ascertainment strategy and is not interpretable as a single prevalence. The individual estimates behind each stratum are plotted in Figure S1, where the spread within a group can be read directly against its pooled value.

The between-group difference was larger for ascertainment strategy (Q = 57.6, df = 2) than for device modality (Q = 13.9, df = 2) or supplementation policy. All three sensitivity analyses left the pattern unchanged, with no group moving by more than 2.4 percentage points and the three ascertainment groups by less than 0.4 (File S1).

### 3.8. Risk of Bias

Sixty-six studies contributed estimates assessed with the JBI prevalence checklist, 12 with ROBINS-I, 4 with RoB 2 and 4 with the JBI case-series checklist; 2 pooled publications were not appraised separately. In studies assessed with ROBINS-I, the main concerns were confounding and selection of participants, with 11 domain-level judgments critical and 25 serious, and an overall judgment of critical in 9 of the 12 appraisals. The largest LCIG trial [72], for example, was at low risk of bias for randomization and missing data, with some concerns about deviations from the intended intervention. It was at high risk for outcome measurement, because the published methods do not describe a systematic assessment of peripheral nerve function.

## 4. Discussion

### 4.1. Summary of Evidence

This systematic review offers an insight into the published studies addressing peripheral neuropathy in patients with Parkinson’s disease treated with device-assisted therapies. The pooled frequency under LCIG was 13.2%, with a cumulative incidence of new-onset PN of 13.7% among patients documented to be free of it beforehand. PN was reported more often in cohorts receiving higher levodopa doses and treated for longer. Under LECIG, the reported number of PN cases remains very low: one case has been reported among 272 patients, apparently the first published case in a LECIG-treated patient. Two further cases were described in a cohort for which the relevant denominator could not be established. Patients with PN had lower vitamin B12 and folate than patients without it in the same cohorts. Neither COMT inhibition nor prophylactic B-vitamin supplementation could be shown to protect against PN, and neither can be excluded.

Our LCIG figure sits between the 42.1% reported by Romagnolo and colleagues [11] and the 1.1% derived from the GLORIA registry’s serious adverse-event code [10]. The difference reflects which studies each draws upon: the higher published figures come from studies designed to detect PN, the lower ones from safety monitoring in trials and registries. Previous reviews have made the same observation [11,12,13,14]; the present analysis quantifies it, at 3.9% where PN was recorded only if spontaneously reported against 33.6% where every patient underwent NCS. Neither figure represents the prevalence expected in routine practice; both are strongly influenced by how PN was assessed.

This issue is further complicated by how safety outcomes are reported and synthesized. A recent systematic review and meta-analysis of subcutaneous levodopa therapies did not evaluate PN as a distinct safety outcome, and its adverse-event synthesis drew on 217 participants from four studies against 483 contributing to the analysis of OFF-time [104]. The individual trials reported adverse events only when they exceeded pre-specified frequency thresholds, set at 5% in the two largest studies. PN occurred less often than this, at 1.2% with ND0612 and 2.7% with foslevodopa–foscarbidopa, and was therefore not captured in those summary tables. Rare adverse events may therefore be missed in general safety summaries even when they are present in the primary trial data. As a result, the evidence available for efficacy is considerably broader than that available for uncommon safety outcomes such as PN. A similar limitation is seen in observational cohorts: the largest published real-world series of subcutaneous foslevodopa–foscarbidopa reported several adverse events in 53 patients, including skin reactions, psychosis, falls and nausea, but did not report any peripheral nerve outcomes [105].

The association between PN and levodopa exposure was consistent across levodopa-equivalent daily dose, levodopa dose and treatment duration. Each association is modest, but the direction is uniform and matches findings in orally treated patients, in whom higher doses and longer exposure have repeatedly been associated with PN [2,11]. Individual case reports point the same way, several describing PN appearing during dose escalation. This is compatible with a dose-dependent iatrogenic mechanism, although an alternative reading warrants consideration. Patients on higher doses for longer are further into their disease, peripheral nerve degeneration occurs in Parkinson’s disease independently of treatment, and disease duration was itself associated with reported PN in our analysis. Peripheral pathology in Parkinson’s disease has recently been framed as causal, parallel, or secondary to central neurodegeneration, with treatment exposure belonging to the third category [16]. What these data cannot do is place device-associated neuropathy in one of those categories rather than another. The metabolic and the neurodegenerative readings need not exclude each other, and mitochondrial dysfunction is one point at which they may meet. In experimental models of Parkinson’s disease, homocysteine aggravated the oxidative stress and mitochondrial dysfunction caused by other toxic insults in dopaminergic cells [106]. In the peripheral nerve, mitochondrial dysfunction has been proposed as a common cause of distal symmetrical sensory neuropathies of toxic and metabolic origin, with degeneration first apparent in intraepidermal nerve fibers [107]. Disrupted mitochondrial homeostasis has also been proposed as a route by which TAR DNA-binding protein 43 (TDP-43) injures neurons in Alzheimer’s disease, another proteinopathy [108]. A metabolic load imposed by treatment could therefore compound a vulnerability that the disease already carries, rather than replace it.

Levodopa exposure and disease progression are difficult to disentangle in observational cohorts. Patients with longer disease duration have usually received levodopa for longer and at higher doses, so univariable models such as those in Table 2 cannot show which of the two carries the association. A study-level meta-regression is poorly placed to resolve this. Its covariates are cohort averages; not every cohort reports both, and the relation between them across cohorts need not mirror the relation within patients. Prospective cohorts could separate the two in several ways. Cumulative levodopa exposure can be modeled as a time-varying covariate in a time-to-event analysis of new-onset PN, with disease duration entered alongside it. Patients matched for disease duration but differing in dose, or in the time since device initiation, provide a direct contrast. So does a concurrent comparison with patients of similar disease duration who remain on oral levodopa. Individual patient data pooled across existing cohorts would allow the same analyses without a new study.

Should levodopa methylation be responsible for raising homocysteine and depleting B vitamins, COMT inhibition ought to reduce the burden on that pathway. This has been reported in orally treated patients by Cossu and colleagues [7] and by Grofik and colleagues [8], and argued for LECIG [9]. Adding a COMT inhibitor lowered homocysteine and raised vitamin B12 in both studies that tried it, in one case under randomization and with supplementation held constant. Neither, however, had enough neuropathy events to show an effect on the nerve. The same conclusion has been drawn from the randomized trial elsewhere [6], and COMT inhibition already appears as an investigational prophylactic measure in a published monitoring protocol for LCIG [5]. Across cohorts, the proportion of patients taking a COMT inhibitor was associated with a lower reported frequency, though the interval includes no effect. LECIG cohorts, in which entacapone is delivered continuously, report both less neuropathy and falling homocysteine. The convergence of these observations is therefore suggestive rather than confirmatory, because the different lines of evidence are not independent: cohorts with greater COMT inhibitor exposure also tend to be more recent, more briefly followed, and less intensively screened for neuropathy. The same pattern appears outside the device literature and with enough events to be seen clearly. In 197 orally treated patients, neuropathy was present in 19.4% of those on levodopa alone against 5.7% of those also taking entacapone. The entacapone group also had lower homocysteine and higher vitamin B12 despite a higher daily levodopa dose [7]. That vitamin B12 rises under COMT inhibition without any change in intake suggests that inhibiting COMT lessens the methylation load itself rather than compensating for the vitamin consumption it produces, which would call for a different preventive strategy.

Patients who had PN had lower vitamin B12 and folate than patients who did not, with no heterogeneity between the three cohorts reporting the comparison. Yet, giving the vitamins prophylactically was not associated with less PN, either overall or within the cohorts that tested every patient, and eight of the individually described patients developed PN while already taking them. A deficiency that accompanies neuropathy is not thereby shown to cause it, and the doses, routes and durations of supplementation vary too much across these studies for their absence of effect to be read as a negative trial. Centers that supplement routinely are also largely those that test nerves routinely, which confounds the comparison further. A frequency that includes neuropathy present before treatment cannot show whether supplementation prevents it. Restricting the comparison to new-onset cases gave the same result, but on six estimates in each group and with very wide intervals. The groups also differ in the regimens used, in baseline vitamin status (reported inconsistently) and in the intensity of surveillance; none of these differences could be adjusted for. Rispoli and colleagues [80] reported a lower incidence under prophylactic multivitamin supplementation, results described as positive yet preliminary by Romagnolo and colleagues [11]; our findings do not settle the matter either way. The vitamin and homocysteine values are already being collected in these cohorts; reporting them separately for patients with and without neuropathy would close the gap without any new measurement.

The low reported frequency of PN with LECIG is noteworthy, but three explanations compete to account for it, and the present data cannot choose between them. The first is surveillance. Most LECIG cohorts relied on passive reporting, and only five of the 272 patients contributing to the estimate underwent nerve conduction studies, whereas systematic testing was more common in the LCIG literature. The second is follow-up. The LECIG cohorts are recent and were followed for shorter periods than most LCIG cohorts, while the median latency to onset among described cases was six months (Section 3.6). A short observation window would therefore miss some cases even when surveillance is adequate. The third is a genuine effect of continuous COMT inhibition, consistent with the falling homocysteine reported in both LECIG cohorts that measured it (Section 3.4). The first two would lower the reported frequency whether or not the third is true. The LECIG and LCIG frequencies therefore cannot be compared directly as measures of risk, and the LECIG figure is not evidence of protection. Until recently, no PN cases had been reported in LECIG-treated cohorts. More recent reports have described one case in the ELEGANCE registry [101] and two additional cases in a Romanian single-center cohort [73]. The Romanian cohort and the ELEGANCE registry may also include overlapping patients, given the shared center and authorship. If so, the identification of two cases in the 50-patient single-center cohort, compared with one case in the 167-patient multinational registry, may reflect more active case finding rather than a true difference in PN frequency. A LECIG cohort assessed by protocol, with NCS and homocysteine before treatment and at fixed intervals for at least 12 months, and with 3-O-methyldopa measured alongside the nerve outcome, would settle a question that now rests on indirect comparison. The metabolite has been quantified in device-treated patients. Müller and colleagues measured levodopa, 3-O-methyldopa and total homocysteine in 28 patients on duodenal levodopa–carbidopa gel, finding homocysteine well above the usual threshold and a correlation of R = 0.7 between levodopa and 3-O-methyldopa [3]. They subsequently showed that methyl-group-donating vitamins raise 3-O-methyldopa in orally and duodenally treated patients [4]. Under LECIG, plasma 3-O-methyldopa falls by approximately 35% relative to LCIG [9]. What has not been done is to measure the metabolite and a peripheral nerve outcome in the same patients.

In the included study to perform serial skin biopsies [48], all five patients developed epidermal denervation within three months, while only one of the four who continued had an abnormal nerve conduction study at twelve months. The sample is small, but the direction of the discrepancy deserves attention. It raises the possibility that NCS underestimates peripheral nerve involvement, and that a figure derived from systematic neurophysiology, such as 33.6%, may not capture the full extent of small-fiber pathology. The wider literature is consistent with this, in that skin biopsy demonstrates reduced intraepidermal nerve fiber density in a high proportion of Parkinson’s disease patients examined [11], although that work has been conducted almost entirely in orally treated patients. Apart from the serial-biopsy study, no included study used quantitative sensory testing, and none used corneal confocal microscopy or sudomotor testing, so these methods have rarely been applied in device-treated cohorts. Future protocols could add to nerve conduction studies a small-fiber measure, such as skin biopsy with intraepidermal nerve fiber density, quantitative sensory testing, corneal confocal microscopy, or sudomotor testing. Small-fiber involvement could then be detected before large-fiber damage is established. Validated diagnostic criteria for small-fiber neuropathy combine clinical signs with quantitative sensory testing and skin biopsy [109].

Focal mononeuropathies were excluded from this review as a distinct entity, but they may add to the neuropathic burden of patients on device-assisted therapy. Two excluded reports describe them after deep brain stimulation. Carpal tunnel syndrome developed in 9 of 115 patients within two years of subthalamic stimulation, a finding the authors explained chiefly by pre-existing symptoms revealed once motor signs, tremor and dyskinesia improved [21]. A spinal accessory neuropathy was thought to result from stretch of the nerve during tunneling of the stimulator extension [110]. The infusion therapies could increase vulnerability to compression in two ways. Weight loss or undernutrition, recorded alongside PN in 11 of the 57 individually described patients (Section 3.6), has been associated with compressive peroneal neuropathy at the fibular head [111]. A nerve already affected by a subclinical polyneuropathy may also be more susceptible to compression, as the double crush hypothesis proposes [112]. Our search was not designed to identify focal neuropathies, so their frequency under device-assisted therapy remains unknown.

PN appeared early, half of the described cases within six months and three quarters within one year, with acute and subacute presentations accounting for 24/40 classifiable cases. This proportion exceeds both the 35.7% reported for LCIG by Romagnolo and colleagues [11] and the 13.6% to 35% range given in a recent review [6]. The authors of that review argue that longitudinal cohorts detect these forms inconsistently and that the true figure is probably lower. All three estimates are based on individually described cases and are therefore vulnerable to reporting bias, particularly the preferential reporting of acute or severe presentations. Our 60% estimate should consequently be regarded as a likely upper bound rather than the true proportion of acute or subacute PN. The device was withdrawn or interrupted in 11 of 12 acute cases, in 8 of 12 subacute cases and in none of the eight chronic cases, a gradient in the same direction as that described by those authors. Among the 46 patients with a documented outcome, 37 improved, stabilized or resolved. Improvement following withdrawal is suggestive of a treatment relationship, but it derives from selected case descriptions in which recoveries are more likely to be published than non-recoveries, and it does not establish causation.

PN clusters within the first year, and baseline nerve status is recorded in only a minority of studies. A monitoring schedule already exists for one of these therapies: vitamin B12, homocysteine and methylmalonic acid before LCIG is started, at six months and yearly thereafter, with neurophysiology before starting and yearly listed as optional [5]. This schedule has been endorsed in a recent review [6]. Our data bear on it in two ways. They support assessing the nerve as well as the biochemistry before the device is initiated, since pre-existing PN cannot otherwise be distinguished from treatment-associated PN. This matters because baseline nerve status was recorded in so few of these studies that cumulative incidence could be estimated from only 24 of 176 estimates. Our data also argue that the first reassessment at six months is late: half of the described cases had appeared by then. A visit at around three months would precede the interquartile range of the observed latency (4 to 12 months), and the acute presentations argue for prompt reassessment whenever sensory symptoms appear at any point. This is a refinement suggested by the latency distribution observed here, not a validated interval, and it is offered for discussion.

### 4.2. Limitations

The limitations fall into two groups: those arising from the included studies and those introduced by our own methodological decisions.

Among the limitations of the included evidence, the main concern is study design. The included studies are overwhelmingly observational and single-arm; four randomized trials contribute and none was designed to detect a difference in PN between arms, which precludes conclusions about causality. Certainty of the evidence was not assessed with the Grading of Recommendations Assessment, Development and Evaluation (GRADE) approach, because the evidence is almost entirely observational and single-arm and the outcome is a reported frequency rather than a comparative effect. Heterogeneity remained high within every group, so the ascertainment strategy accounts for much of the variation between studies but not all of it. The residual variation may reflect differences in case definition, nerve selection, normative reference data and genuine clinical difference, which these data cannot separate.

A further limitation arises from our synthesis approach. The primary summary measure combines point prevalence, period prevalence and adverse-event frequency, and because these are distinct estimands, the pooled value should be interpreted as a reported frequency rather than as an estimate of prevalence.

Prevalent and incident PN could not be separated in most studies, baseline status being rarely recorded. Diabetes mellitus and other competing causes were reported inconsistently, which prevented adjustment. Small-fiber involvement, severity scales and time-to-event data are each reported in too few studies to synthesize. Five of the eleven overlap clusters rest on probable rather than certain overlap. Overlap was classified as probable where it was inferred from a shared center, shared investigators and overlapping recruitment periods rather than stated by the authors. Where a report was withheld and the overlap was in fact absent, a study was excluded unnecessarily. In two clusters, two reports were retained despite possible overlap, so some patients may be counted twice (Table S3).

The supplementation analysis compares policies rather than regimens. The included studies differ in the route of administration (oral or intramuscular), in dose, in preparation and in when supplementation began relative to device initiation. These details were not extracted as variables, so they could not be analyzed. The differences may matter. Absorption of oral cobalamin depends on intrinsic factor and ileal uptake, or at high doses on passive diffusion of a small fraction of the dose, whereas intramuscular administration bypasses the gut [113]. On low-quality evidence, high-dose oral and intramuscular cobalamin appear similarly effective in correcting serum levels [114], but that evidence does not come from patients on continuous intestinal levodopa, whose gastrointestinal function and nutritional state may differ. An absence of effect across heterogeneous regimens therefore does not show that no regimen would be effective.

Classifying the ascertainment strategy of each study required judgment, because no validated framework exists for this purpose. The groupings were therefore taken from the assessment procedures the studies themselves describe, each estimate being coded from the methods section rather than from the results. Contestable codings were adjudicated independently against the sources and five were revised. Coding from the described procedure limits the scope for a study’s findings to influence where it is placed without removing it entirely.

Finally, publication and reporting bias cannot be excluded. The more relevant bias here may be the non-reporting of an outcome that was measured. Two eligible cohorts performed NCS in every patient and reported the result only as a severity score or a nerve conduction parameter, without a case count that could enter the synthesis.

## 5. Conclusions and Future Perspectives

The reported frequency of peripheral neuropathy in patients on device-assisted therapy depends heavily on how systematically it is assessed, ranging from 3.9% where it is recorded only when reported spontaneously to 33.6% where every patient undergoes nerve conduction studies. Because reported PN frequency varies substantially with ascertainment, the pooled LCIG estimate of 13.2% should be interpreted as a summary across studies using different detection strategies, rather than as a single estimate of the true prevalence of PN.

Greater levodopa exposure and longer treatment were associated with more reported neuropathy, consistently across measures but modestly, and these observational data cannot separate treatment exposure from disease progression. Neither COMT inhibition nor prophylactic B-vitamin supplementation could be shown to protect against PN; both remain plausible and untested, and patients with neuropathy did have lower vitamin B12 and folate than those without.

Peripheral nerve function should be assessed before a device-assisted therapy is started, so that pre-existing neuropathy is not later attributed to treatment. Since half of the described cases appeared within six months, these findings also support early reassessment during the first year, with three months a reasonable interval for prospective evaluation rather than an established recommendation.

What the field needs is a prospective cohort with protocolized neurophysiology, documented baseline nerve status and small-fiber testing, reporting biochemical values separately for patients with and without neuropathy.

Such a cohort can answer the clinical question but cannot establish mechanism. The pathways discussed here, levodopa methylation, homocysteine and B-vitamin handling, and mitochondrial homeostasis, can now be perturbed one gene at a time in cellular and animal models with current genome-editing tools [115,116]. Experiments of that kind could test causally what observational data can only associate.

## Figures and Tables

**Table 1:** Reported frequency of peripheral neuropathy by device modality. *k*, number of estimates contributing; *n*/*N*, PN cases over patients assessed; CI, confidence interval. Rows with *k* = 1 are single-study frequencies with a Wilson interval, not pooled estimates. The LECIG figure is likewise the aggregate proportion with a Wilson interval rather than a pooled estimate, for the reason given below, as is the apomorphine figure, which aggregates two discordant arms (0/39 and 4/4). Two further cases, reported by a cohort whose denominator could not be established, are excluded from the LECIG figure and are discussed in Section 3.3. The subthalamic deep brain stimulation estimate rests on two arms of very unequal size and its interval spans almost the entire range; it is not a stable estimate. n.a., not applicable, used where no pooled estimate was calculated.

Device	*k*	*n*/*N*	% (95% CI)	I^2^
LCIG	42	403/3853	13.2 (9.8 to 17.6)	85%
LECIG	4	1/272	0.4 (0.1 to 2.1)	n.a.
Subthalamic deep brain stimulation	2	3/106	8.7 (0.0 to 96.3)	91%
Foslevodopa-foscarbidopa	1	2/74	2.7 (0.7 to 9.3)	n.a.
Levodopa–carbidopa, subcutaneous (ND0612)	1	4/322	1.2 (0.5 to 3.2)	n.a.
Apomorphine, subcutaneous	2	4/43	9.3 (3.7 to 21.6)	n.a.

**Table 2:** Association between the reported frequency of peripheral neuropathy and study-level covariates, by univariable random-effects meta-regression on the logit scale. Odds ratios are per the unit shown. *k* is the number of contributing estimates. These models are exploratory.

Covariate	*k*	Odds Ratio	95% CI	*p*
Levodopa-equivalent daily dose, per 100 mg/day	25	1.22	1.00 to 1.49	0.052
Levodopa dose, per 100 mg/day	20	1.28	1.05 to 1.55	0.012
Duration of device treatment, per 12 months	45	1.30	1.10 to 1.54	0.002
Disease duration, per year	42	1.16	1.04 to 1.28	0.006
Patient age, per year	42	1.15	1.02 to 1.29	0.017
Patients on a COMT inhibitor, per 10 percentage points	21	0.90	0.74 to 1.08	0.256
Publication year, per year	52	1.02	0.94 to 1.11	0.605
Patients assessed, per 100 patients	52	0.77	0.61 to 0.96	0.019

**Table 3:** Vitamin B12, homocysteine and folate in patients with peripheral neuropathy against patients without, within the same cohort. Differences are expressed as the value in patients with PN minus the value in patients without, pooled by a random-effects model on the difference scale. One study reports medians with quartiles, from which a mean and standard deviation were approximated. In two studies, the samples were drawn before device initiation [55,80], in the third at the time PN was diagnosed [65].

Analyte	*k*	Mean Difference (95% CI)	I^2^	*p*
Vitamin B12, pg/mL	3	−81.5 (−120.2 to −42.9)	0%	<0.001
Folate, ng/mL	3	−1.27 (−2.37 to −0.18)	0%	0.023
Homocysteine, µmol/L	3	+6.2 (−4.3 to +16.7)	84%	0.245

**Table 4:** Reported frequency of peripheral neuropathy by B-vitamin supplementation policy. *k* is the number of contributing estimates, not of studies: an estimate enters only if it survives the cohort-overlap rule and reports more than one patient. Only the first and third rows are compared with each other, for the reasons given in the text.

Supplementation Policy	*k*	n/N	Pooled % (95% CI)	I^2^
Prophylactic, from treatment initiation	8	162/1076	26.4 (13.8 to 44.4)	86%
Deficiency-triggered	5	30/117	25.1 (12.8 to 43.4)	66%
None	11	99/597	22.7 (13.0 to 36.7)	81%
Not reported	28	126/2880	5.0 (3.2 to 7.8)	80%

**Table 5:** Reported frequency of peripheral neuropathy by PN ascertainment strategy. *k* is the number of contributing estimates. Rows marked not pooled rest on a single study and are shown descriptively; n.a., not applicable, used where no pooled estimate was calculated. Heterogeneity remains high within the pooled groups (I^2^ 67–78%), so the difference between them should not be attributed to the ascertainment strategy alone. The between-group test compares the three strategies with more than a handful of contributing patients; the other two rest on 10 and four patients and are given descriptively.

PN Ascertainment Strategy	*k*	n/N	Pooled % (95% CI)	I^2^
Recorded only if spontaneously reported	27	93/2861	3.9 (2.6 to 5.7)	67%
Examined or tested when symptoms appeared	9	219/1409	20.3 (14.5 to 27.6)	78%
All patients examined, no neurophysiology	2	8/10	not pooled	n.a.
NCS in all patients	13	96/386	33.6 (20.8 to 49.3)	78%
NCS plus small-fiber testing in all	1	1/4	not pooled	n.a.

## Data Availability

All data analyzed in this review were extracted from published reports, each of which is cited. The extracted counts, the study characteristics and the workbook containing the full analysis are provided as Supplementary Materials (Table S7 and File S1). Every pooled estimate, subgroup test and meta-regression reported in this paper is computed by live formulas in that workbook from the extracted counts, and the figures are drawn from the values it produces. The review protocol was registered with PROSPERO on 21 April 2026 (CRD 1375888) before data extraction began, with no amendments after extraction started other than the post hoc analyses identified as such in Section 2.6 and Section 3.3.

## Supplementary Materials

The following supporting information can be downloaded at: https://www.mdpi.com/article/10.3390/biomedicines14102279/s1. Table S1: PRISMA 2020 checklist; Table S2: Search strategy; Table S3: Cohort-overlap clusters; Table S4: Corrections applied after a second reviewer re-read the sources; Table S5: Risk-of-bias judgments, one row per appraisal and one column per checklist item; Table S6: Reports excluded after full-text assessment, each with its category and reason; Table S7: Characteristics of the 75 included studies, in alphabetical order; Table S8: What each biochemical marker can support; Figure S1: Study-level forest plot of every estimate contributing to the three pooled ascertainment strata; File S1: Analysis workbook. References [117,118,119,120,121,122,123,124,125,126,127,128,129,130,131,132,133,134,135,136,137,138,139,140,141,142,143,144,145,146,147,148,149,150,151] are cited in the supplementary materials.

## Abbreviations

The following abbreviations are used in this manuscript:

Abbreviation	Meaning
CI	confidence interval
COMT	catechol-O-methyltransferase
JBI	Joanna Briggs Institute
LCIG	levodopa–carbidopa intestinal gel
LECIG	levodopa-entacapone-carbidopa intestinal gel
NCS	nerve conduction studies
PN	peripheral neuropathy
