# Supplementary material for: Peripheral Neuropathy in Parkinson’s Disease Treated with Device-Assisted Therapies: How Often It Is Reported Depends on How It Is Sought. A Systematic Review and Meta-Analysis
Source: Biomedicines. 2026 Oct 8;14(10):2279. doi: 10.3390/biomedicines14102279 (PMC13701721; doi:10.3390/biomedicines14102279)
Supplement: Supplementary file 1 [file biomedicines-14-02279-s001.zip › Figure_S1.pdf]

## Supplementary Material

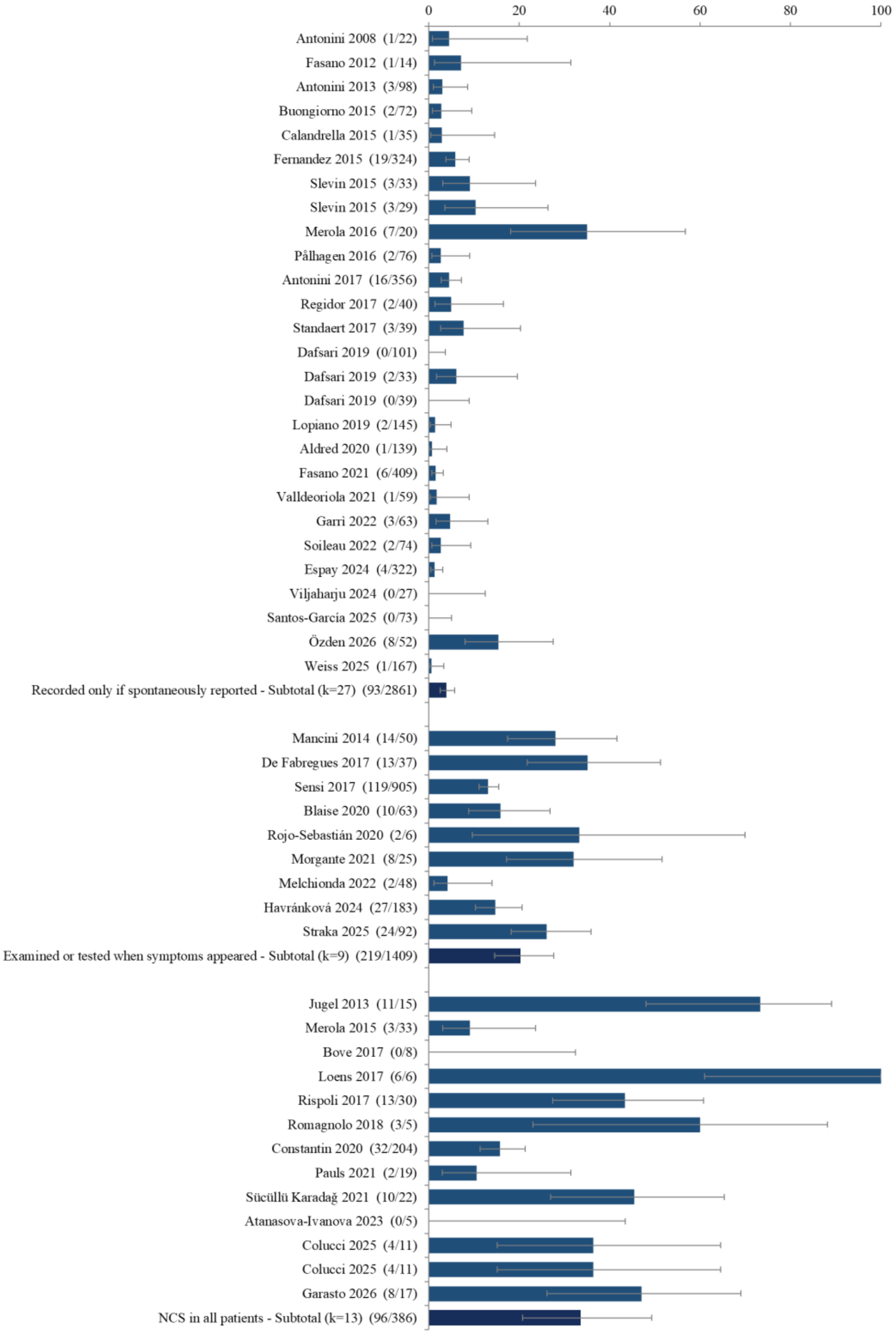


**Figure S1.** Study-level forest plot of every estimate contributing to the three pooled ascertainment strata, drawn from the values on sheet 7 of File S1. Each bar is the reported percentage of patients with peripheral neuropathy in one arm, with the number of cases over the number assessed given beside the study; the horizontal line is the Wilson 95% confidence interval for that single estimate. The darker bar at the foot of each stratum is the pooled random-effects estimate for that stratum, with its confidence

interval, and carries the number of contributing estimates and the aggregate counts. Estimates are listed in the order of sheet 1 of File S1. Forty-nine of the 52 reported-frequency estimates are shown: the remaining three belong to the two strategies that rest on 10 and 4 patients and are reported descriptively rather than pooled, as set out in Table 5. Where an arm reported no cases the bar has zero length and only the interval is visible. The chart was produced in Microsoft Excel from the workbook supplied as File S1. Study labels are those of Table S7.
