# Supplementary material for: Peripheral Neuropathy in Parkinson’s Disease Treated with Device-Assisted Therapies: How Often It Is Reported Depends on How It Is Sought. A Systematic Review and Meta-Analysis
Source: Biomedicines. 2026 Oct 8;14(10):2279. doi: 10.3390/biomedicines14102279 (PMC13701721; doi:10.3390/biomedicines14102279)
Supplement: Supplementary file 1 [file biomedicines-14-02279-s001.zip › Table_S1.pdf]

Supplementary Material

Table S1. PRISMA 2020 checklist. The last column gives the section, table, figure or supplementary file of this review that addresses each item.

#	Item	Checklist item	Where addressed
1	Title	Identify the report as a systematic review	Title
2	Abstract	See the PRISMA 2020 for Abstracts checklist	Abstract
3	Rationale	Describe the rationale in the context of existing knowledge	1
4	Objectives	Provide an explicit statement of the objectives	1.2
5	Eligibility criteria	Specify the inclusion and exclusion criteria	2.2
6	Information sources	Specify all sources searched and the date of the last search	2.3, Table S2
7	Search strategy	Present the full search strategy for all sources	Table S2
8	Selection process	Specify the methods used to decide inclusion	2.3
9	Data collection process	Specify the methods used to collect data	2.4
10	Data items	List and define all outcomes and other variables sought	2.4
11	Study risk of bias assessment	Specify the methods used to assess risk of bias	2.5
12	Effect measures	Specify the effect measure used for each outcome	2.6
13	Synthesis methods	Describe the processes and methods used to synthesize results	2.6, File S1
14	Reporting bias assessment	Describe the methods used to assess reporting bias	4.2
15	Certainty assessment	Describe the methods used to assess certainty	4.2 (narrative; GRADE not applied)
16	Study selection	Report the numbers screened, assessed and included	3.1, Figure 1
17	Study characteristics	Cite each included study and present its characteristics	3.1, Table S7
18	Risk of bias in studies	Present the risk-of-bias assessments	3.8, Table S5
19	Results of individual studies	Present the summary statistics for each study	Table S7, File S1, Figure S1

20	Results of syntheses	Present the results of every synthesis	3.2 to 3.7, Tables 1 to 5
21	Reporting biases	Present the assessment of risk of bias due to missing results	4.2
22	Certainty of evidence	Present the assessment of certainty	4.2
23	Discussion	Interpretation, limitations and implications	4
24	Registration and protocol	Give the registration name and number	2.1; Data Availability Statement
25	Support	Describe the sources of financial and non-financial support	Funding
26	Competing interests	Declare any competing interests	Conflicts of Interest
27	Availability of data, code and other materials	Report which materials are publicly available	Data Availability, File S1

Items 15 and 22 are addressed narratively. GRADE was not applied, because the included evidence is almost entirely observational and single-arm and the outcome is a reported frequency rather than a comparative effect; the reasons are set out in Section 4.2.
