# Supplementary material for: Peripheral Neuropathy in Parkinson’s Disease Treated with Device-Assisted Therapies: How Often It Is Reported Depends on How It Is Sought. A Systematic Review and Meta-Analysis
Source: Biomedicines. 2026 Oct 8;14(10):2279. doi: 10.3390/biomedicines14102279 (PMC13701721; doi:10.3390/biomedicines14102279)
Supplement: Supplementary file 1 [file biomedicines-14-02279-s001.zip › Table_S2.pdf]

Supplementary Material

Table S2. Search strategy. The same concept structure was used in each database: a Parkinson's disease concept, a device-assisted therapy concept and a peripheral nervous system concept, combined with AND. No date, language or publication-type limits were applied.

Database	Interface	Date run	Records
PubMed	pubmed.ncbi.nlm.nih.gov	22 April 2026	525
Scopus	scopus.com	22 April 2026	352
Web of Science	webofscience.com, Core Collection	22 April 2026	131
Total			1008
Manual screening of 21 topic reviews	reference lists	15 August 2026	8 reports, plus 1 from another source

PubMed

```
("Parkinson Disease"[Mesh] OR "Parkinson*"[tiab] OR "parkinsonism"[tiab]) AND ("levodopa-carbidopa intestinal gel"[tiab] OR "LCIG"[tiab] OR "LECIG"[tiab] OR "Duodopa"[tiab] OR "Duopa"[tiab] OR "Lecigon"[tiab] OR "intrajejunal"[tiab] OR "intraduodenal"[tiab] OR "foslevodopa"[tiab] OR "foscarbidopa"[tiab] OR "ND0612"[tiab] OR "apomorphine infusion"[tiab] OR "subcutaneous apomorphine"[tiab] OR "Deep Brain Stimulation"[Mesh] OR "deep brain stimulation"[tiab] OR "subthalamic"[tiab] OR "device-aided"[tiab] OR "device-assisted"[tiab]) AND ("Peripheral Nervous System Diseases"[Mesh] OR "Polyneuropathies"[Mesh] OR "polyneuropath*"[tiab] OR "neuropath*"[tiab] OR "nerve conduction"[tiab] OR "electromyograph*"[tiab] OR "intraepidermal nerve fib*"[tiab] OR "small fib*"[tiab])
```

Scopus

```
TITLE-ABS-KEY(("Parkinson*" OR parkinsonism) AND ("levodopa-carbidopa intestinal gel" OR LCIG OR LECIG OR Duodopa OR Duopa OR Lecigon OR intrajejunal OR intraduodenal OR foslevodopa OR foscarbidopa OR ND0612 OR "apomorphine infusion" OR "deep brain stimulation" OR subthalamic OR "device-aided" OR "device-assisted") AND (polyneuropath* OR neuropath* OR "nerve conduction" OR electromyograph* OR "intraepidermal nerve fibre" OR "intraepidermal nerve fiber" OR "small fibre" OR "small fiber"))
```

Web of Science, Core Collection

```
TS=("Parkinson*" OR parkinsonism) AND TS=("levodopa-carbidopa intestinal gel" OR LCIG OR LECIG OR Duodopa OR Duopa OR Lecigon OR intrajejunal OR intraduodenal OR foslevodopa OR foscarbidopa OR ND0612 OR "apomorphine infusion" OR "deep brain stimulation" OR subthalamic OR "device-aided" OR "device-assisted") AND TS=(polyneuropath* OR neuropath* OR "nerve conduction" OR electromyograph* OR "intraepidermal nerve fib*" OR "small fib*")
```

In addition, the reference lists of 21 topic reviews were screened manually, which yielded eight further reports; one more was identified from another source, and all nine were assessed in full text. Manual screening continued after the database searches and the last review was screened on 15 August 2026. Two items therefore postdate the database search: one included study, published on 23 April 2026, and one of

the topic reviews, available online on 1 July 2026. Both entered through manual screening, which PRISMA treats as a separate source with its own date.

The protocol was registered with PROSPERO on 21 April 2026, the day before the database searches were run.
