# Supplementary material for: Peripheral Neuropathy in Parkinson’s Disease Treated with Device-Assisted Therapies: How Often It Is Reported Depends on How It Is Sought. A Systematic Review and Meta-Analysis
Source: Biomedicines. 2026 Oct 8;14(10):2279. doi: 10.3390/biomedicines14102279 (PMC13701721; doi:10.3390/biomedicines14102279)
Supplement: Supplementary file 1 [file biomedicines-14-02279-s001.zip › Table_S3.pdf]

Supplementary Material

Table S3. Cohort-overlap clusters. Where two or more reports describe the same patients, one was retained for the pooled analyses and the others were withheld, except where the selection rule states otherwise. *Certainty* is *certain* where the authors state the overlap or trial registration numbers match, and *probable* where it was inferred from a shared center, shared investigators and overlapping recruitment periods. References are numbered as in the manuscript. Study labels give the year of first online publication, which for three studies (Klostermann 2011, Lang 2015 and Merola 2015) is one year earlier than the year of the issue given in the reference list.

Cluster	Reports	Retained	Withheld	Certainty	Selection rule
PIVOTAL	Olanow 2014 [72]; Slevin 2015 [90]	Slevin 2015 [90]	Olanow 2014 [72]	Certain	Slevin 2015 is the open-label extension of the double-blind trial reported by Olanow 2014 and covers the same patients over a longer exposure. The trial itself is described narratively (Sections 3.3 and 3.8).
GLORIA	Antonini 2017 [10]; Poewe 2019 [77]; Antonini 2020 [34]; Szász 2020 [95]	Antonini 2017 [10]	Poewe 2019 [77]; Antonini 2020 [34]; Szász 2020 [95]	Certain	Antonini 2017 is the final report of the registry. The other three are a post hoc analysis of the same safety population and two national subsets contained in it.
COSMOS	Fasano 2021 [52]; Simu 2021 [89]	Fasano 2021 [52]	Simu 2021 [89]	Certain	Simu 2021 is the Romanian subanalysis of the multinational study reported by Fasano 2021.
PHASE3	Fernandez 2015 [53]; Lang 2015 [61]; Fernandez 2018 [54]; Zadikoff 2020 [102]	Fernandez 2015 [53]	Lang 2015 [61]; Fernandez 2018 [54]; Zadikoff 2020 [102]	Certain (trial registratio n numbers)	Lang 2015 pools 412 patients from four trials of the phase 3 program, including those reported by Fernandez 2015 and Slevin 2015; Fernandez 2018 reports the extension of one of them; Zadikoff 2020 pools the same program with the GLORIA registry. Fernandez 2015, an original trial report, was retained; the two pooled publications and the extension report were withheld.
TORINO	Merola 2014 [68]; Zibetti 2014 [103]; Merola 2015 [69]; Artusi 2020 [35]	Merola 2015 [69]	Merola 2014 [68]; Zibetti 2014 [103]; Artusi 2020 [35]	Certain	Reports from the same center describing overlapping patients. Merola 2015 was retained: it used the same nerve conduction work-up as Merola 2014, assessed more patients and covers a later recruitment window.
ANTONINI-EARLY	Antonini 2007 [31]; Antonini 2008 [32]	Antonini 2008 [32]	Antonini 2007 [31]	Certain	Antonini 2008 contains the nine patients of Antonini 2007.
FERRARA	Sensi 2014 [87]; Rispoli 2017 [80]	Rispoli 2017 [80]	Sensi 2014 [87]	Probable	Same center, with recruitment overlapping in 2011 to 2013; the report with more patients assessed was retained.

BARCELONA	Buongiorno 2015 [40]; De Fabregues 2017 [47]	Buongiorno 2015 [40]; De Fabregues 2017 [47]	None	Probable	Overlap is possible but unconfirmed and at most partial: De Fabregues 2017 comes from one of the five centers contributing to Buongiorno 2015. The two fall in different ascertainment strata and are never compared with each other, but both enter the LCIG and all-strata pooled estimates, where any shared patients would be counted twice. Both were retained, with the possible overlap declared.
BERLIN	Klostermann 2011 [60]; Jugel 2013 [58]	Jugel 2013 [58]	Klostermann 2011 [60]	Probable	Same center and corresponding author, consistent with one continuously recruiting program. Jugel 2013 performed nerve conduction studies in every LCIG patient and reports exposure; Klostermann 2011 tested only symptomatic patients.
BRATISLAVA	Valkovič 2022 [96]; Havránková 2024 [57]	Havránková 2024 [57]	Valkovič 2022 [96]	Probable	Possible overlap between the two cohorts. Havránková 2024 is much the larger and reports a case count; Valkovič 2022 reports no case count.
LECIG-ELEGANCE	Weiss 2025 [101]; Orbán-Kis 2026 [73]; Atanasova-Ivanova 2023 [36]	Weiss 2025 [101]; Atanasova-Ivanova 2023 [36]	Orbán-Kis 2026 [73]	Probable	Weiss 2025 (ELEGANCE registry) states its denominator explicitly and was retained. Orbán-Kis 2026 was withheld because the size of the subset containing its two cases is not stated, so no denominator can be derived, and because its cohort probably overlaps the registry through a shared center and investigators. Atanasova-Ivanova 2023 was retained because it falls in a different ascertainment stratum and at most five patients could be shared.

Reports withheld from the pooled analyses because another report of the same patients was used: 16. They are marked as overlap in Table S7.
