# Supplementary material for: Peripheral Neuropathy in Parkinson’s Disease Treated with Device-Assisted Therapies: How Often It Is Reported Depends on How It Is Sought. A Systematic Review and Meta-Analysis
Source: Biomedicines. 2026 Oct 8;14(10):2279. doi: 10.3390/biomedicines14102279 (PMC13701721; doi:10.3390/biomedicines14102279)
Supplement: Supplementary file 1 [file biomedicines-14-02279-s001.zip › Table_S4.pdf]

Supplementary Material

Table S4. Corrections applied after a second reviewer re-read the sources. Each entry records the field corrected, the value first extracted, the value retained and the reason. Strategy labels are those of Table S7.

Study	Record	Field	Extracted	Retained	Reason
Jugel 2013	comparator arm	Patients with sensory abnormality only	10	5	The same nesting error as in the LCIG arm (next row).
Jugel 2013	LCIG arm	Patients with sensory abnormality only	15	5	The reported counts are nested, not additive: ten patients had sensory abnormality and five of them had motor abnormality as well, so summing the two gave 15, more than the 11 patients with neuropathy in the arm.
Bohlega 2015	LCIG arm	Denominator	inferred	reported directly	The denominator of 18 is stated directly in the source; no inference was needed.
Fernandez 2015	estimate	Source of the count	table	computed by reviewer	No integer counts are printed except for serious adverse events; numerators were derived from percentages and carry rounding uncertainty.
Chang 2016	estimate	Ascertainment strategy	NCS all	if sympt.	The earlier coding rested only on 'NCS' appearing in a figure legend describing the care pathway. The text describes work-up of patients who presented with symptoms, not screening of the cohort.
Chang 2016	estimate	Population screened	all patients	symptomatic only	Follows from the recoding above.
Merola 2016	estimate	Ascertainment strategy	if sympt.	spontaneous	No polyneuropathy assessment of any kind is described; the clinical description refers to cases once found, not to a method of finding them.
Juhász 2017	estimate	Ascertainment strategy	if sympt.	spontaneous	The paper states that electroneurography was not performed, uses no polyneuropathy scale and gives no case definition; the two cases appear in a table column headed 'Preferred term'. Reports of this kind are coded as spontaneous elsewhere in the review.
Rispoli 2017	new-onset estimate	Subclinical abnormality only	not recorded	4	The four patients removed from the numerator (next row) are recorded here.
Rispoli 2017	new-onset estimate	PN cases	4	0	The four incident cases are stated three times to be asymptomatic and without clinical signs. Subclinical electrophysiological abnormality alone was not counted as PN, so the new-onset estimate is 0/21. The four patients remain in the prevalence estimate (13/30), whose case definition rests on nerve conduction studies.

Sensi 2017	estimate	Source of the count	table	computed by reviewer	The table prints percentages only; the integer numerators were back-computed from one-decimal percentages of 905.
Fernandez 2018	estimate	Source of the count	table	computed by reviewer	Per-term counts are not published, so the numerator is bracketed rather than read from a table.
Constantin 2020	estimate	Source of the count	table	main text	The value appears in the Results text, not in a table.
Rojo-Sebastián 2020	LCIG arm, clinical estimate	Used in the pool	yes	no	The permissive estimate (3/6) comes from a row labeled 'clinical data suggestive of polyneuropathy' that includes numbness and tingling, which were not counted as PN.
Rojo-Sebastián 2020	LCIG arm, confirmed estimate	Used in the pool	no	yes	The electrophysiologically confirmed estimate (2/6) becomes the pooled row.
Rojo-Sebastián 2020	comparator arm, clinical estimate	Used in the pool	yes	no	Same reasoning as for the LCIG arm.
Rojo-Sebastián 2020	comparator arm, confirmed estimate	Used in the pool	no	yes	Same reasoning as for the LCIG arm (0/18 replaces 5/18).
Straka 2025	estimate	Ascertainment strategy	all exam.	if sympt.	The methods state that nerve conduction studies were performed only in patients presenting clinical symptoms of polyneuropathy, which defines this strategy, and every counted case was confirmed by nerve conduction studies.
Özden 2026	estimate	Ascertainment strategy	if sympt.	spontaneous	Nerve conduction studies and electromyography are not mentioned anywhere in the paper; the 'distal sensorimotor' descriptor characterizes cases, not a method of ascertainment.

Total corrections: 19.
