# Supplementary material for: Peripheral Neuropathy in Parkinson’s Disease Treated with Device-Assisted Therapies: How Often It Is Reported Depends on How It Is Sought. A Systematic Review and Meta-Analysis
Source: Biomedicines. 2026 Oct 8;14(10):2279. doi: 10.3390/biomedicines14102279 (PMC13701721; doi:10.3390/biomedicines14102279)
Supplement: Supplementary file 1 [file biomedicines-14-02279-s001.zip › Table_S5.pdf]

Supplementary Material

Table S5. Risk-of-bias judgments, one row per appraisal and one column per checklist item. Appraisal was at the level of the estimate, so a study contributing more than one kind of estimate is appraised more than once and appears in more than one panel. Column headings are the checklist item numbers; the items themselves are listed under each panel.

Judgments are abbreviated as Y (yes), N (no), U (unclear) and n.a. (not applicable) for the JBI checklists, and given in full for RoB 2 and ROBINS-I. NR means that no judgment was recorded for that item. The column Estimate appraised gives the outcome that the appraisal addressed, as recorded by the appraiser, and need not match the estimand label of Table S7; overall means that the appraisal applies to the study as a whole. Abbreviations: AE, adverse event; JBI, Joanna Briggs Institute; PN, peripheral neuropathy.

S5.1. JBI critical appraisal checklist for studies reporting prevalence data (66 appraisals)

Study	Estimate appraised	1	2	3	4	5	6	7	8	9	Overall
Aldred 2020	as AE	Y	U	Y	N	Y	N	N	N	U	high
Antonini 2007	incidence	Y	N	N	Y	Y	U	N	N	Y	NR
Antonini 2008	as AE	Y	U	N	N	Y	N	N	N	Y	high
Antonini 2013	as AE	Y	Y	Y	Y	Y	N	N	Y	U	high
Antonini 2017	as AE	Y	U	Y	Y	Y	N	N	Y	U	high
Antonini 2020	as AE	Y	Y	U	Y	Y	N	N	Y	U	high
Artusi 2020	prevalence	Y	Y	Y	Y	Y	Y	N	Y	U	NR
Atanasova-Ivanova 2023	prevalence	Y	Y	N	U	N	U	Y	N	N	NR
Blaise 2020	prevalence	Y	Y	Y	Y	Y	N	N	Y	Y	NR
Bohlega 2015	incidence	Y	Y	N	Y	Y	N	U	N	Y	NR
Bove 2017	prevalence	Y	Y	N	U	Y	Y	Y	U	Y	moderate
Buongiorno 2015	as AE	Y	U	Y	Y	Y	N	N	U	U	high
Cáceres-Redondo 2014	incidence	Y	Y	N	Y	Y	Y	N	U	U	NR
Calandrella 2015	as AE	Y	Y	N	Y	Y	N	N	N	N	high
Chang 2016	incidence	Y	Y	N	Y	Y	U	U	N	Y	NR
Colucci 2025	prevalence	N	Y	N	Y	Y	N	Y	N	U	NR
Constantin 2020	prevalence	Y	Y	Y	N	Y	Y	U	Y	U	NR
Dafsari 2019	prevalence	Y	Y	N	Y	Y	N	N	N	Y	high
De Fabregues 2017	prevalence	Y	U	N	Y	U	U	N	N	U	high
Devigili 2016	prevalence	Y	N	N	N	Y	Y	Y	U	Y	high
Espay 2024	prevalence	Y	N	Y	U	Y	N	U	U	N	high
Fasano 2012	as AE	Y	Y	N	Y	Y	N	N	N	Y	high
Fasano 2021	as AE	N	U	Y	Y	Y	N	N	U	U	high
Fernandez 2015	as AE	Y	U	Y	Y	Y	N	N	N	U	NR
Fernandez 2018	as AE	N	Y	Y	Y	Y	N	N	N	N	NR
Garasto 2026	prevalence	Y	Y	N	U	U	Y	Y	U	Y	low
Garrì 2022	prevalence	Y	Y	U	Y	N	N	N	N	U	high
Havránková 2024	prevalence	Y	Y	Y	N	Y	U	N	Y	U	moderate
Jugel 2013	prevalence	Y	N	N	Y	N	Y	Y	U	U	high
Juhász 2017	incidence	Y	Y	N	Y	Y	N	N	Y	Y	high
Klostermann 2011	prevalence	Y	U	N	N	U	Y	N	N	n.a.	NR
Loens 2017	prevalence	N	N	N	Y	N	Y	Y	N	N	high
Lopiano 2019	prevalence	Y	Y	Y	Y	Y	N	N	U	Y	NR
Mancini 2014	prevalence	Y	Y	N	U	Y	Y	N	Y	U	NR
Melchionda 2022	prevalence	Y	Y	N	Y	Y	U	N	N	U	high
Meppelink 2011	incidence	Y	U	N	Y	Y	U	N	N	Y	NR
Merola 2014	prevalence	Y	Y	N	Y	N	Y	Y	U	N	NR
Merola 2015	overall	Y	Y	N	Y	Y	Y	Y	Y	Y	NR
Merola 2016	prevalence	N	N	N	Y	Y	N	N	N	U	NR

Morgante 2021	prevalence	Y	N	N	Y	N	Y	U	Y	U	high
Orbán-Kis 2026	PN frequency	Y	Y	U	Y	N	N	N	N	U	NR
Özden 2026	incidence	Y	Y	N	Y	Y	N	N	N	U	NR
Pålhagen 2016	prevalence	Y	U	N	Y	Y	N	N	N	U	NR
Pauls 2021	incidence	Y	Y	N	Y	U	Y	Y	U	U	NR
Poewe 2019	as AE	Y	N	N	Y	N	N	N	N	N	NR
Pürner 2023	as AE	N	N	N	Y	U	N	N	N	N	NR
Regidor 2017	as AE	U	U	N	Y	Y	N	N	N	Y	high
Rispoli 2017	prevalence	Y	Y	N	Y	U	Y	Y	N	U	moderate
Rojo-Sebastián 2020	prevalence	N	Y	N	U	Y	N	N	Y	U	high
Romagnolo 2018	prevalence	Y	Y	N	Y	Y	U	Y	N	Y	NR
Rus 2022	incidence	Y	Y	Y	N	Y	Y	N	Y	U	NR
Sajin 2025	prevalence	N	Y	Y	Y	Y	U	N	N	Y	moderate
Santos-García 2025	as AE	Y	Y	N	Y	Y	N	N	N	U	high
Sensi 2014	prevalence	Y	Y	N	U	U	Y	U	N	U	moderate
Sensi 2017	prevalence	Y	U	Y	N	U	U	N	N	Y	NR
Simu 2021	prevalence	N	N	N	Y	N	N	N	N	U	high
Slevin 2015	as AE	N	Y	N	Y	Y	N	Y	Y	Y	high
Standaert 2017	as AE	Y	U	N	Y	Y	N	U	U	Y	high
Straka 2025	prevalence	Y	Y	Y	N	Y	Y	U	Y	U	moderate
Sücüllü Karadağ 2021	prevalence	Y	Y	N	Y	U	Y	U	U	Y	moderate
Szász 2020	prevalence	Y	U	N	Y	Y	N	N	N	Y	high
Valldeoriola 2021	as AE	Y	U	N	Y	Y	N	N	N	Y	high
Viljaharju 2022	incidence	Y	Y	Y	Y	Y	N	N	U	U	moderate
Viljaharju 2024	prevalence	Y	Y	N	Y	N	N	N	N	N	high
Weiss 2025	PN frequency	Y	U	Y	Y	U	N	N	Y	Y	NR
Zibetti 2014	as AE	Y	Y	N	Y	Y	N	N	N	Y	high

Items: **1** Was the sample frame appropriate to address the target population; **2** Were study participants recruited in an appropriate way; **3** Was the sample size adequate; **4** Were the study subjects and the setting described in detail; **5** Was the data analysis conducted with sufficient coverage of the identified sample; **6** Were valid methods used for the identification of the condition; **7** Was the condition measured in a standard, reliable way for all participants; **8** Was there appropriate statistical analysis; **9** Was the response rate adequate, and if not, was the low response rate managed appropriately.

S5.2. JBI critical appraisal checklist for case series (4 appraisals)

**Study**	**Estimate appraised**	**1**	**2**	**3**	**4**	**5**	**6**	**7**	**8**	**9**	**10**	**Overall**
Klostermann 2011	incidence	N	Y	Y	U	Y	Y	Y	Y	U	n.a.	NR
Lehnerer 2014	incidence	N	N	U	U	U	N	U	Y	Y	n.a.	high
Santos-García 2012	incidence	N	N	U	N	N	Y	U	Y	N	n.a.	high
Vial-Daragon 2026	incidence	N	Y	Y	U	N	Y	Y	U	U	n.a.	NR

Items: **1** Were there clear criteria for inclusion in the case series; **2** Was the condition measured in a standard, reliable way for all participants included in the case series; **3** Were valid methods used for identification of the condition for all participants included in the case series; **4** Did the case series have consecutive inclusion of participants; **5** Did the case series have complete inclusion of participants; **6** Was there clear reporting of the demographics of the participants in the study; **7** Was there clear reporting of clinical information of the participants; **8** Were the outcomes or follow up results of cases clearly reported; **9** Was there clear reporting of the presenting site(s)/clinic(s) demographic information; **10** Was statistical analysis appropriate.

S5.3. ROBINS-I (12 appraisals)

**Study**	**Estimate appraised**	**1**	**2**	**3**	**4**	**5**	**6**	**7**	**Overall**

Dafsari 2019	as AE	serious	moderate	low	moderate	moderate	critical	moderate	critical
Devigili 2016	incidence	critical	serious	low	moderate	moderate	moderate	serious	critical
Erdem 2025	overall	serious	serious	moderate	moderate	moderate	moderate	serious	serious
Garasto 2026	overall	serious	low	moderate	moderate	serious	low	moderate	serious
Jugel 2013	overall	serious	serious	low	moderate	low	moderate	moderate	serious
Loens 2017	prevalence	critical	critical	moderate	moderate	serious	moderate	moderate	critical
Mancini 2014	prevalence	critical	moderate	low	low	moderate	moderate	moderate	critical
Merola 2016	as AE	critical	moderate	low	moderate	moderate	critical	moderate	critical
Regidor 2017	overall	critical	serious	low	serious	moderate	serious	serious	critical
Rojo-Sebastián 2020	prevalence	critical	serious	low	moderate	serious	serious	moderate	critical
Sajin 2025	overall	critical	moderate	serious	moderate	serious	serious	serious	critical
Valkovič 2022	biomarker	critical	serious	low	serious	moderate	moderate	serious	critical

Items: **1** Bias due to confounding; **2** Bias in selection of participants into the study; **3** Bias in classification of interventions; **4** Bias due to deviations from intended interventions; **5** Bias due to missing data; **6** Bias in measurement of outcomes; **7** Bias in selection of the reported result.

S5.4. RoB 2 (4 appraisals)

**Study**	**Estimate appraised**	**1**	**2**	**3**	**4**	**5**	**Overall**
Colucci 2025	incidence	some concerns	some concerns	low	low	some concerns	high
Espay 2024	as AE	low	some concerns	low	some concerns	low	some concerns
Olanow 2014	as AE	low	some concerns	low	high	some concerns	high
Soileau 2022	as AE	low	some concerns	low	high	some concerns	high

Items: **1** Bias arising from the randomization process; **2** Bias due to deviations from intended interventions; **3** Bias due to missing outcome data; **4** Bias in measurement of the outcome; **5** Bias in selection of the reported result.

S5.5. Not formally appraised (2 appraisals)

**Study**	**Estimate appraised**	**1**	**Overall**
Lang 2015	overall	n.a.	n.a.
Zadikoff 2020	overall	n.a.	n.a.

Items: 1 Overall: not appraised (pooled publication superseded by its primary reports).

Appraisals: 88 across 75 studies, covering 796 recorded judgments at item and summary level.

A summary judgment across the whole checklist was not recorded for 30 of the appraisals shown above. The item-level judgments for those studies are complete; only the summary line is absent, and it is shown as NR rather than derived after the fact. No analysis in the review depends on it: the risk-of-bias narrative in Section 3.8 reports item-level judgments, and no estimate was weighted or excluded by overall rating.
