# Supplementary material for: Peripheral Neuropathy in Parkinson’s Disease Treated with Device-Assisted Therapies: How Often It Is Reported Depends on How It Is Sought. A Systematic Review and Meta-Analysis
Source: Biomedicines. 2026 Oct 8;14(10):2279. doi: 10.3390/biomedicines14102279 (PMC13701721; doi:10.3390/biomedicines14102279)
Supplement: Supplementary file 1 [file biomedicines-14-02279-s001.zip › Table_S6.pdf]

Supplementary Material

Table S6. Reports excluded after full-text assessment, each with its category and reason. Categories were applied in priority order, so every report carries the single most decisive reason. The category totals are those shown in Figure 1.

Category	Rule	n
C	No device-assisted therapy in the population	23
B	No peripheral nerve outcome reported in the cohort	11
A	Fewer than four patients (case report or series of three or fewer)	8
D	Mononeuropathy or post-surgical Guillain-Barré	3
E	Not primary data (review, editorial or letter without original cases)	2
	**Total**	**47**

#	Report	Category	Reason
1	Adewusi JK, Hadjivassiliou M, Vinagre-Aragón A, O'Connor KR, Khan A, Grünewald RA, Zis P. 2018. Acta Neurol Belg, doi:10.1007/s13760-018-0947-3. "Sensory neuropathic symptoms in idiopathic Parkinson's disease: prevalence and impact on quality of life."	C	No device-assisted therapy in the cohort.
2	Andréasson M, Brodin L, Laffita-Mesa JM, Svenningsson P. 2017. J Parkinsons Dis 7:619–628. "Correlations between methionine cycle metabolism, COMT genotype, and polyneuropathy in L-dopa treated Parkinson's disease: a preliminary cross-sectional study."	C	The studied exposure is oral levodopa dose; the five LCIG patients are an unanalyzed covariate, not a device cohort.
3	Ceravolo R, Cossu G, Bandettini di Poggio M, Santoro L, Barone P, Zibetti M, Frosini D, Nicoletti V, Manganelli F, Iodice R, Picillo M, Merola A, Lopiano L, et al. 2013. Mov Disord 28(10):1391–1397. "Neuropathy and levodopa in Parkinson's disease: evidence from a multicenter study."	C	Oral-levodopa exposure study; no device-treated arm.
4	Chen Q, Scherbaum R, Gold R, Pitarokoili K, Mosig A, Zella S, Tönges L. 2023. J Neural Transm 130:763–776. "Data-driven subtyping of Parkinson's disease: comparison of current methodologies and application to the Bochum PNS cohort."	C	No device-assisted therapy; a subtyping-methods paper in which PN scores are input variables.
5	Conradt C, Guo D, Miclea A, Nisslein T, Ismail C, Chatamra K, Andersohn F. 2018. J Parkinsons Dis 8:141–144. "Increased prevalence of polyneuropathy in Parkinson's disease patients: an observational study."	C	Pre-treatment PD; no antiparkinsonian therapy, let alone device therapy.
6	Cossu G, Ceravolo R, Zibetti M, Arca R, Ricchi V, Paribello A, Murgia D, Merola A, Romagnolo A, Nicoletti V, Palermo G, Mereu A, Lopiano L, Melis M, Abbruzzese G, Bonuccelli U. 2016. Parkinsonism Relat Disord 27:81–84 (Short communication). "Levodopa and neuropathy risk in patients with Parkinson disease: effect of COMT inhibition."	C	Oral levodopa ± oral COMT inhibitor; no device therapy.
7	Grambalová Z, Kaiserová M, Vaštík M, Menšíková K, Otruba P, Zapletalová J, Dufek J, Kaňovský P. 2015. Neuroendocrinol Lett 36(4):363–367. "Peripheral neuropathy in Parkinson's disease."	C	Oral levodopa only; no device therapy in the cohort.
8	Grofik M, Sivák Š, Nosáľ V, Turčanová Koprušáková M, Michalik J, Čierny D, Tatarková Z, Kurča E. 2018. J Neurol Sci, doi:10.1016/j.jns.2018.06.012. "The influence	C	The authors excluded LCIG patients by protocol.

	of levodopa, entacapone and homocysteine on prevalence of polyneuropathy in patients with Parkinson's disease."		
9	Kass-Iliyya L, Javed S, Gosal D, Kobylecki C, Marshall A, Petropoulos IN, Ponirakis G, Tavakoli M, Ferdousi M, Chaudhuri KR, Jeziorska M, Malik RA, Silverdale MA. 2015. Parkinsonism Relat Disord 21(12):1454–1460. "Small fiber neuropathy in Parkinson's disease: a clinical, pathological and corneal confocal microscopy study."	C	No device-assisted therapy in the cohort.
10	Kimber TE, Blumbergs PC, Thompson PD. 2013. Parkinsonism Relat Disord 19:847–849 (Letter). "Severe ataxic polyneuropathy associated with chronic levodopa use in Parkinson's disease."	C	Four patients, meeting the size threshold, but the exposure is high-dose oral levodopa with no device therapy.
11	Lee JJ, Baik JS. 2020. Yonsei Med J 61(12):1050–1053. "Peripheral neuropathy in de novo patients with Parkinson's disease."	C	Drug-naïve PD; no antiparkinsonian therapy, let alone device therapy.
12	Liu N, Zhang J. 2024. Ideggyogy Sz 77(5–6):161–166. "Study on the relationship between peripheral nerve fiber types and levodopa usage in Parkinson's disease."	C	Oral levodopa or drug-naïve; no device therapy, and only 11 exposed patients.
13	Müller T, Renger K, Kuhn W. 2004. Arch Neurol 61:657–660. "Levodopa-associated increase of homocysteine levels and sural axonal neurodegeneration."	C	Oral levodopa homocysteine study; no device therapy.
14	Nolano M, Provitera V, Manganelli F, Iodice R, Stancanelli A, Caporaso G, Saltalamacchia A, Califano F, Lanzillo B, Picillo M, Barone P, Santoro L. 2017. Neurology 89(8):776–784. "Loss of cutaneous large and small fibers in naive and L-dopa–treated PD patients."	C	No device-assisted therapy; an oral-versus-naïve comparison.
15	Park J-S, Park D, Ko P-W, Kang K, Lee H-W. 2017. Neurol Sci, doi:10.1007/s10072-017-3056-9. "Serum methylmalonic acid correlates with neuropathic pain in idiopathic Parkinson's disease."	C	Oral levodopa cohort; no device therapy.
16	Rajabally YA, Martey J. 2013. Acta Neurol Scand 127:216–220, doi:10.1111/ane.12011. "No association between neuropathy and restless legs in Parkinson's disease."	C	No device therapy; PN is a covariate in a study about restless legs.
17	Rajabally YA, Martey J. 2013. J Neurol 260(11):2844–2848. "Levodopa, vitamins, ageing and the neuropathy of Parkinson's disease."	C	Oral/levodopa-naïve comparison; no device therapy.
18	Szadejko K, Dziewiatowski K, Szabat K, Robowski P, Schinwelski M, Sitek E, Sławek J. 2016. J Neurol Sci, doi:10.1016/j.jns.2016.09.061. "Polyneuropathy in levodopa-treated Parkinson's patients."	C	Oral levodopa-treated cohort with DBS excluded by protocol; no device subgroup.
19	Toth C, Breithaupt K, Ge S, Duan Y, Terris JM, Thiessen A, Wiebe S, Zochodne DW, Suchowersky O. 2010. Ann Neurol 68:28–36. "Levodopa, methylmalonic acid, and neuropathy in idiopathic Parkinson disease."	C	Oral levodopa exposure study with brain surgery explicitly absent.
20	Toth C, Brown MS, Furtado S, Suchowersky O, Zochodne D. 2008. Mov Disord 23(13):1850–1859. "Neuropathy as a potential complication of levodopa use in Parkinson's disease."	C	Oral levodopa cohort; DBS/pallidotomy is recorded but never analyzed as an exposure.
21	Xie C, Gan R, Chen J, Li Y, He P, Gao Y, Wang L, Ma G, Zhang Y, Nie K, Wang L. 2025. Parkinsonism Relat Disord 135:107842. "Clinical and electrophysiological	C	Oral levodopa only; DBS excluded by the authors' protocol.

	characteristics of large and small fiber neuropathy in Parkinson's disease."		
22	Xiong Y-T, Liu M-H, Gu H-Y, Li K, Zhang J-R, Cheng X-Y, Jin H, Chen J, Mao C-J, Liu C-F. 2022. Acta Neurol Scand 146:75–81. "Fiber selectivity of peripheral neuropathy in patients with Parkinson's disease."	C	Oral levodopa exposure strata; no device therapy.
23	de Araújo DF, de Melo Neto AP, Oliveira ÍSC, Brito BS, de Araújo IT, Barros IS, Lima JWO, Horta WG, Gondim FAA. 2016. BMC Neurol 16:139. "Small (autonomic) and large fiber neuropathy in Parkinson disease and parkinsonism."	C	No device therapy; the population also dilutes PD with other parkinsonisms.
24	Boyd JT, Zadikoff C, Benesh JA, Zamudio J, Robieson WZ, Kukreja P, Yokoyama M, Siddiqui MS. 2020. Clin Park Relat Disord 2:25–34. "A post hoc comparison of levodopa-carbidopa intestinal gel daytime monotherapy vs polytherapy safety and efficacy in patients with advanced Parkinson's disease: results from 6 phase 3/3b open-label studies."	B	No peripheral nerve outcome reported for the cohort.
25	Freire-Alvarez E, Kurča E, Lopez Manzanares L, Pekkonen E, Spanaki C, Vanni P, Liu Y, Sanchez-Soliño O, Barbato LM. 2021. Mov Disord 36(11):2615–2623. "Levodopa-carbidopa intestinal gel reduces dyskinesia in Parkinson's disease in a randomized trial." (DYSCOVER, NCT02799381)	B	An LCIG randomized trial whose endpoint is dyskinesia and which reports no peripheral-nerve outcome of any kind.
26	Ikenaka K, Kajiyama Y, Aguirre C, Choong C-J, Taniguchi S, Doi J, Wang N, Ajiki T, Ogawa K, Kakuda K, Kimura Y, Mochizuki H. 2024. Pharmacol Res Perspect 12:e1174. "Decreased hepatic enzymes reflect the decreased vitamin B6 levels in Parkinson's disease patients."	B	A B6/liver-enzyme biomarker study in LCIG patients with no peripheral-nerve outcome.
27	Müller T, Jugel C, Ehret R, Ebersbach G, Bengel G, Muhlack S, Klostermann F. 2011. J Neural Transm 118:1329–1333 (Short communication). "Elevation of total homocysteine levels in patients with Parkinson's disease treated with duodenal levodopa/carbidopa gel."	B	LCIG homocysteine/3-OMD study with no nerve assessment.
28	Müller T, Jugel C, Muhlack S, Klostermann F. 2013. Clin Neuropharmacol 36(2):52–54. "Methyl group–donating vitamins elevate 3-O-methyldopa in patients with Parkinson disease."	B	LCIG 3-OMD/homocysteine study with no nerve assessment.
29	Rukavina K, Ebersbach G, Gruber D. 2025. Mov Disord Clin Pract 12:2317–2323. "Foslevodopa/foscarbidopa continuous subcutaneous infusion in Parkinson's disease: real-world short-term data on tolerability, infusion rate adjustments and concomitant medication."	B	No peripheral nerve outcome of any kind is reported.
30	Szász JA, Dulamea AO, Constantin VA, Mureşanu DF, Dumbravă LP, Tiu C, Jianu DC, Simu M, et al. 2024. Am J Ther 31:e209–e218. "Levodopa-carbidopa-entacapone intestinal gel in advanced Parkinson disease: a multicenter real-life experience."	B	No peripheral nerve outcome reported for the 74 LECIG patients.
31	Szatmári S, Szász JA, Orbán-Kis K, Baróti B, Bataga S, Ciorba M, Nagy EE, Neagoe RM, Mihály I, Szász PZ, Kelemen K, Frigy A, Szilveszter M, Constantin VA. 2024. Pharmaceutics 16(4):453. "Levodopa–entacapone–carbidopa intestinal gel in the treatment of advanced	B	LECIG motor-outcome series with no peripheral-nerve outcome.

	Parkinson's disease: a single center real-world experience."		
32	Öthman M, Bergquist F, Odin P, Scharfenort M, Johansson A, Markaki I, Svenningsson P, Dizdar N, Nyholm D. 2025. Eur J Neurol 32:e16582. "Levodopa–entacapone–carbidopa intestinal gel: data from the Swedish national registry for Parkinson's disease."	B	LECIG registry cohort with no peripheral-nerve outcome.
33	Öthman M, Nyholm D. 2024. Mov Disord Clin Pract 11(12):1609–1612 (Brief report). "A 4-year follow-up of levodopa-entacapone-carbidopa intestinal gel treatment in Parkinson's disease."	B	LECIG retention and mortality report with no peripheral-nerve outcome.
34	Öthman M, Widman E, Nygren I, Nyholm D. 2021. J Pers Med 11(4):254. "Initial experience of the levodopa–entacapone–carbidopa intestinal gel in clinical practice."	B	LECIG usability/tolerability report with no peripheral-nerve outcome.
35	Cochez H, Cazenave C, Castagnet C, Balguerie L, Acket B, Fabbri M, Ory-Magne F. 2026. Mov Disord 41(5):1317–1318. "Acute inflammatory demyelinating polyradiculoneuropathy-like associated with subcutaneous foslevodopa–foscarbidopa: first report."	A	Single case; below the size threshold for case series.
36	Galazky I, Schoof J, Stallforth S, Kupsch A, Heinze H-J, Kluge C. 2014. Parkinsonism Relat Disord 20(1):125–127 (Letter). "Guillain–Barré/CIDP-like neuropathy in two parkinsonian patients following intestinal levodopa/carbidopa treatment."	A	Two cases; below the size threshold for case series.
37	Kaur H, Bhatia K, Yelam A, Shukla A, Asher I, Govindarajan R. 2018. Cureus 10(7):e3055. "Infusion-rate-dependent acute neuropathic pain with Duopa in a patient with Parkinson's disease and pre-existing neuropathy."	A	Single case; below the size threshold for case series.
38	Pintér D, Deli G, Juhász A, Pál E, Janszky J, Kovács N. 2019. Eur J Neurol 26(12):e96–e97 (Letter). "Long-term successful treatment of chronic inflammatory demyelinating polyneuropathy-like polyneuropathy induced by levodopa-carbidopa intestinal gel with intravenous immunoglobulin."	A	Single case; below the size threshold for case series.
39	Rivero-de-Aguilar A, Sesar Á, Fernández-Pajarín G, Ares-Pensado B, Castro A. 2019. Rev Neurol 69(7):304–306 (Spanish; Correspondence). "Polirradiculoneuropatía desmielinizante inflamatoria crónica asociada al tratamiento con infusión intraduodenal de levodopa-carbidopa."	A	Single case; below the size threshold for case series.
40	Ueno T, Baba M, Haga R, Arai A, Hattori N, Tomiyama M. 2024. Neurol Clin Neurosci 12(3):195–197. "Sensory neuropathy complicated with de novo diabetes mellitus during levodopa-carbidopa intestinal gel infusion in a patient with Parkinson's disease: a case report."	A	Single case; below the size threshold for case series.
41	Urban PP, Wellach I, Faiss S, Layer P, Rosenkranz T, Knop K, Weis J. 2010. Mov Disord 25(11):1748–1752 (Brief Report). "Subacute axonal neuropathy in Parkinson's disease with cobalamin and vitamin B6 deficiency under Duodopa therapy."	A	Two cases; below the size threshold for case series.
42	Valli A, Scheperjans F. 2025. Mov Disord 40:2538–2539. "Subacute painful axonal polyneuropathy associated with	A	Single case report.

	foslevodopa/foscarbidopa subcutaneous infusion in advanced Parkinson's disease."		
43	Loizon M, Laurencin C, Vial C, Danaila T, Thobois S. 2016. J Neurol 263:2416–2418, doi:10.1007/s00415-016-8279-9. "High incidence of carpal tunnel syndrome after deep brain stimulation in Parkinson's disease."	D	Compressive median mononeuropathy confirmed electrodiagnostically, not a generalized distal symmetric polyneuropathy.
44	Samii A, Slimp JC, Friedly JL, Goodkin R. 2007. Stereotact Funct Neurosurg 85:296–298. "Spinal accessory neuropathy after deep brain stimulation for Parkinson's disease."	D	Isolated mechanical mononeuropathy of the spinal accessory nerve after tunneling of the stimulator extension.
45	Theuriet J, Aguesse C, Bouhour F, Jomir L, Thobois S, Prange S. 2024. Rev Neurol (Paris) 180:459–461. "Guillain-Barré syndrome following subthalamic nucleus deep brain stimulation in Parkinson's disease: a case report."	D	A post-surgical, iatrogenically triggered event, mechanistically distinct from the pathway this review addresses.
46	Onofrj M, Bonanni L, Cossu G, Manca D, Stocchi F, Thomas A. 2009. Parkinsonism Relat Disord 15S3:S233–S236. "Emergencies in parkinsonism: akinetic crisis, life-threatening dyskinesias, and polyneuropathy during L-Dopa gel treatment."	E	A narrative review with no original patient data.
47	Uncini A, Eleopra R, Onofrj M. 2015. J Neurol Neurosurg Psychiatry 86(5):490–495 (online 2014), doi:10.1136/jnnp-2014-308586. "Polyneuropathy associated with duodenal infusion of levodopa in Parkinson's disease: features, pathogenesis and management."	E	A review, not an eligible primary study design; its single new index case would also fall below the size threshold.

The 47 reports above reconcile exactly with Figure 1: 44 excluded after full-text assessment of the database search and 3 after citation searching and other sources.
