# Supplementary material for: Peripheral Neuropathy in Parkinson’s Disease Treated with Device-Assisted Therapies: How Often It Is Reported Depends on How It Is Sought. A Systematic Review and Meta-Analysis
Source: Biomedicines. 2026 Oct 8;14(10):2279. doi: 10.3390/biomedicines14102279 (PMC13701721; doi:10.3390/biomedicines14102279)
Supplement: Supplementary file 1 [file biomedicines-14-02279-s001.zip › Table_S7.pdf]

Supplementary Material

Table S7. Characteristics of the 75 included studies, in alphabetical order. *Sought* records how the study looked for neuropathy: *spontaneous* = recorded only when spontaneously reported; *if sympt.* = examined or tested once symptoms appeared; *all exam.* = every patient examined, without neurophysiology; *NCS all* = nerve conduction studies in every patient; *NCS+SF* = nerve conduction studies and small-fiber assessment in every patient. *n/N gives every estimate pooled for the study, one per arm; where a study contributes no reported-frequency estimate, it is the cumulative incidence (Estimand: Incidence), which is pooled separately;* studies marked *overlap* (cluster names as in Table S3) are included but withheld from the pooled analyses because another report of the same patients was used. Two studies assessed neuropathy systematically but reported it only as a continuous outcome and supply no count. Study labels give the year of first online publication, which for three studies (Klostermann 2011, Lang 2015 and Merola 2015) is one year earlier than the year of the issue given in the reference list. Country is the primary country of the study (ISO 3166-1 alpha-3 code); an asterisk marks the seven studies that recruited across regions and are counted as multinational in Section 3.1. N is the number of patients assessed for PN in the device arms. Abbreviations: AE, adverse event; APO SC, subcutaneous apomorphine; DBS, deep brain stimulation; DBS-STN, subthalamic deep brain stimulation; FLFC SC, subcutaneous foslevodopa-foscarbidopa; LCIG, levodopa-carbidopa intestinal gel; LECIG, levodopa-entacapone-carbidopa intestinal gel; ND0612 SC, subcutaneous levodopa-carbidopa; PN, peripheral neuropathy; prev., prevalence; RCT, randomized controlled trial; Exposure (mo), device exposure in months; hand search, identified by citation searching or from another source; n.a., not applicable or not reported.

Study	Country	Design	Device	N	Exposure (mo)	Sought	Estimand	n/N	%	Notes
Aldred 2020 [30]	USA	Prospective cohort	LCIG	139	6	spontaneous	AE frequency	1/139	0.7	
Antonini 2007 [31]	ITA	Prospective cohort	LCIG	9	12	spontaneous	Incidence	1/9	11.1	overlap: ANTONINI-EARLY
Antonini 2008 [32]	ITA	Prospective cohort	LCIG	22	24	spontaneous	AE frequency	1/22	4.5	
Antonini 2013 [33]	ITA	Retrospective cohort	LCIG	98	20	spontaneous	AE frequency	3/98	3.1	
Antonini 2017 [10]	ITA*	Registry	LCIG	356	21	spontaneous	AE frequency	16/356	4.5	
Antonini 2020 [34]	ITA	Registry	LCIG	56	24	spontaneous	AE frequency	4/56	7.1	overlap: GLORIA
Artusi 2020 [35]	ITA	Retrospective cohort	LCIG	91	64	if sympt.	Period prev.	10/91	11.0	overlap: TORINO
Atanasova-Ivanova 2023 [36]	BGR	Prospective cohort	LECIG	5	12	NCS all	Point prev.	0/5	0.0	
Blaise 2020 [37]	FRA	Retrospective cohort	LCIG	63	19	if sympt.	Period prev.	10/63	15.9	
Bohlega 2015 [38]	SAU	Prospective cohort	LCIG	18	48	all exam.	Incidence	0/18	0.0	
Bove 2017 [39]	ITA	Prospective cohort	LCIG	8	6	NCS all	Point prev.	0/8	0.0	
Buongiorno 2015 [40]	ESP	Registry	LCIG	72	22	spontaneous	AE frequency	2/72	2.8	
Cáceres-Redondo 2014 [41]	ESP	Retrospective cohort	LCIG	29	32	if sympt.	Incidence	4/29	13.8	
Calandrella 2015 [42]	ITA	Retrospective cohort	LCIG	35	32	spontaneous	AE frequency	1/35	2.9	hand search
Chang 2016 [43]	AUS	Prospective cohort	LCIG	15	12	if sympt.	Incidence	7/15	46.7	
Colucci 2025 [44]	ITA	RCT	LCIG	22	12	NCS all	Point prev.	4/11; 4/11	36.4; 36.4	opicapone and control arms

Constantin 2020 [45]	ROU	Retrospective cohort	LCIG	204	28	NCS all	Period prev.	32/204	15.7	
Dafsari 2019 [46]	DEU	Comparative cohort	DBS-STN, LCIG, APO SC	173	6	spontaneous	AE frequency	0/101; 2/33; 0/39	0.0; 6.1; 0.0	DBS-STN, LCIG and apomorphine arms
De Fabregues 2017 [47]	ESP	Prospective cohort	LCIG	37	44	if sympt.	Period prev.	13/37	35.1	
Devigili 2016 [48]	ITA	Comparative cohort	LCIG	5	12	NCS+SF	Point prev.	1/4	25.0	four of the five patients completed follow-up
Erdem 2025 [49]	TUR	Cross-sectional	LCIG	14	n.a.	NCS all	Continuous	no count	n.a.	
Espay 2024 [50]	USA*	RCT	ND0612 SC	322	4	spontaneous	AE frequency	4/322	1.2	open-label ND0612 population, which includes the 128 later randomized to ND0612
Fasano 2012 [51]	ITA	Retrospective cohort	LCIG	14	25	spontaneous	AE frequency	1/14	7.1	hand search
Fasano 2021 [52]	USA*	Retrospective cohort	LCIG	409	36	spontaneous	AE frequency	6/409	1.5	
Fernandez 2015 [53]	USA*	Prospective cohort	LCIG	324	12	spontaneous	AE frequency	19/324	5.9	
Fernandez 2018 [54]	USA*	Prospective cohort	LCIG	262	49	spontaneous	AE frequency	9/262	3.4	overlap: PHASE3
Garasto 2026 [55]	ITA	Comparative cohort	LCIG	17	40	NCS all	Point prev.	8/17	47.1	
Garrì 2022 [56]	ITA	Retrospective cohort	LCIG	63	48	spontaneous	AE frequency	3/63	4.8	
Havránková 2024 [57]	CZE	Retrospective cohort	LCIG	183	n.a.	if sympt.	Point prev.	27/183	14.8	
Jugel 2013 [58]	DEU	Cross-sectional	LCIG	15	24	NCS all	Point prev.	11/15	73.3	
Juhász 2017 [59]	HUN	Prospective cohort	LCIG	34	12	spontaneous	Incidence	2/34	5.9	
Klostermann 2011 [60]	DEU	Case series	LCIG	20	n.a.	if sympt.	Period prev.	2/20	10.0	overlap: BERLIN
Lang 2015 [61]	USA	Pooled analysis	LCIG	412	30	spontaneous	AE frequency	24/412	5.8	overlap: PHASE3
Lehnerer 2014 [62]	DEU	Case series	LCIG	31	n.a.	if sympt.	Incidence	3/31	9.7	
Loens 2017 [63]	DEU	Cross-sectional	LCIG	6	37	NCS all	Point prev.	6/6	100.0	new-onset PN 2/22 on symptom-triggered work-up
Lopiano 2019 [64]	ITA	Prospective cohort	LCIG	145	37	spontaneous	AE frequency	2/145	1.4	
Mancini 2014 [65]	ITA	Cross-sectional	LCIG	50	34	if sympt.	Point prev.	14/50	28.0	
Melchionda 2022 [66]	ITA	Retrospective cohort	LCIG	48	24	if sympt.	Point prev.	2/48	4.2	

Meppelink 2011 [67]	NLD	Comparative cohort	LCIG	15	6	if sympt.	Incidence	2/8; 0/7	25.0; 0.0	
Merola 2014 [68]	ITA	Prospective cohort	LCIG	10	9	NCS all	Point prev.	4/10	40.0	overlap: TORINO
Merola 2015 [69]	ITA	Prospective cohort	LCIG	33	24	NCS all	Point prev.	3/33	9.1	
Merola 2016 [70]	ITA	Comparative cohort	LCIG	20	62	spontaneous	AE frequency	7/20	35.0	
Morgante 2021 [71]	ITA	Cross-sectional	LCIG	25	59	if sympt.	Period prev.	8/25	32.0	
Olanow 2014 [72]	USA	RCT	LCIG	37	3	spontaneous	AE frequency	1/37	2.7	overlap: PIVOTAL; hand search
Orbán-Kis 2026 [73]	ROU	Retrospective cohort	LECIG	n.a.	n.a.	n.a.	n.a.	n.a.	n.a.	overlap: LECIG-ELEGANCE; hand search
Özden 2026 [74]	TUR	Retrospective cohort	LCIG	52	102	spontaneous	AE frequency	8/52	15.4	
Pålhagen 2016 [75]	SWE	Prospective cohort	LCIG	76	36	spontaneous	AE frequency	2/76	2.6	
Pauls 2021 [76]	FIN	Retrospective cohort	LCIG	19	6	NCS all	Point prev.	2/19	10.5	
Poewe 2019 [77]	AUT	Post hoc	LCIG	208	24	spontaneous	AE frequency	4/81	4.9	overlap: GLORIA; N sums three treatment groups, n/N is the polytherapy group
Pürner 2023 [78]	DEU	Retrospective cohort	n.a.	n.a.	n.a.	spontaneous	AE frequency	n.a.	n.a.	mixed-therapy cohort (1/116), not pooled
Regidor 2017 [79]	ESP	Comparative cohort	LCIG	19	29	spontaneous	AE frequency	2/40	5.0	pooled over all 40 patients on LCIG; N and exposure refer to the 19 who also had DBS
Rispoli 2017 [80]	ITA	Prospective cohort	LCIG	30	42	NCS all	Point prev.	13/30	43.3	
Rojo-Sebastián 2020 [81]	ESP	Cross-sectional	LCIG	6	n.a.	if sympt.	Point prev.	2/6	33.3	
Romagnolo 2018 [82]	ITA	Cross-sectional, descriptive	DBS-STN	5	175	NCS all	Point prev.	3/5	60.0	
Rus 2022 [83]	SVN	Retrospective cohort	LCIG	103	56	NCS all	Incidence	13/103	12.6	
Sajin 2025 [84]	DEU	Cross-sectional	LCIG, APO SC, DBS	11	n.a.	all exam.	Point prev.	4/6; 4/4	66.7; 100.0	LCIG and apomorphine arms; a third arm of one patient is not pooled
Santos-García 2012 [85]	ESP	Case series	LCIG	5	7	n.a.	n.a.	n.a.	n.a.	
Santos-García 2025 [86]	ESP	Retrospective cohort	LECIG	73	6	spontaneous	AE frequency	0/73	0.0	
Sensi 2014 [87]	ITA	Prospective cohort	LCIG	28	32	NCS all	Point prev.	9/28	32.1	overlap: FERRARA

Sensi 2017 [88]	ITA	Survey	LCIG	905	n.a.	if sympt.	AE frequency	119/905	13.1	
Simu 2021 [89]	ROU	Cross-sectional	LCIG	73	32	spontaneous	AE frequency	1/95	1.1	overlap: COSMOS; N is the subset tested by NCS, 1/95 the safety population
Slevin 2015 [90]	USA	Prospective cohort	LCIG	62	13	spontaneous	AE frequency	3/33; 3/29	9.1; 10.3	
Soileau 2022 [91]	USA	RCT	FLFC SC	74	3	spontaneous	AE frequency	2/74	2.7	
Standaert 2017 [92]	USA	Prospective cohort	LCIG	39	14	spontaneous	AE frequency	3/39	7.7	
Straka 2025 [93]	SVK	Retrospective cohort	LCIG	92	30	if sympt.	Period prev.	24/92	26.1	a comparator arm of 14 patients on LECIG, with no case, is not pooled
Sücüllü Karadağ 2021 [94]	TUR	Prospective cohort	LCIG	22	18	NCS all	Point prev.	10/22	45.5	
Szász 2020 [95]	ROU	Registry	LCIG	35	24	spontaneous	AE frequency	5/35	14.3	overlap: GLORIA
Valkovič 2022 [96]	SVK	Comparative cohort	LCIG	16	n.a.	NCS all	Continuous	no count	n.a.	overlap: BRATISLAVA
Valldeoriola 2021 [97]	ESP	Prospective cohort	LCIG	59	6	spontaneous	AE frequency	1/59	1.7	hand search
Vial-Daragon 2026 [98]	FRA	Case series	FLFC SC	4	3	n.a.	n.a.	n.a.	n.a.	
Viljaharju 2022 [99]	FIN	Retrospective cohort	LCIG	103	42	if sympt.	Incidence	4/103	3.9	
Viljaharju 2024 [100]	FIN	Retrospective cohort	LECIG	27	5	spontaneous	AE frequency	0/27	0.0	
Weiss 2025 [101]	DEU*	Registry	LECIG	167	7	spontaneous	AE frequency	1/167	0.6	hand search
Zadikoff 2020 [102]	USA*	Pooled analysis	LCIG	768	28	spontaneous	AE frequency	25/412	6.1	overlap: PHASE3; N is the pooled population (412 phase 3, 356 GLORIA), n/N the phase 3 part
Zibetti 2014 [103]	ITA	Prospective cohort	LCIG	59	26	spontaneous	AE frequency	4/59	6.8	overlap: TORINO
