# Supplementary material for: Peripheral Neuropathy in Parkinson’s Disease Treated with Device-Assisted Therapies: How Often It Is Reported Depends on How It Is Sought. A Systematic Review and Meta-Analysis
Source: Biomedicines. 2026 Oct 8;14(10):2279. doi: 10.3390/biomedicines14102279 (PMC13701721; doi:10.3390/biomedicines14102279)
Supplement: Supplementary file 1 [file biomedicines-14-02279-s001.zip › Table_S8.pdf]

Supplementary Material

Table S8. What each biochemical marker can support. *Measured* counts the included studies that report the analyte in any form. *Numeric value* counts those giving a concentration rather than only a count of patients outside a reference limit. *Compared by PN status* counts those reporting the analyte separately for patients with and without peripheral neuropathy in the same cohort. *Before and during treatment* counts those reporting it at baseline and again on the device for the same group of patients. The last two columns are what the analysis in Section 3.4 rests on; the gap between the first column and the last two is the reporting gap identified in the Discussion. The last column includes reports that give the analyte only for subgroups, for individually described patients or as counts outside a reference range; the cohorts described in Section 3.4 are those with a whole-cohort numeric value at both time points.

Analyte	Measured	Numeric value	Compared by PN status	Before and during treatment
Vitamin B12	25	21	3	7
Homocysteine	19	17	3	7
Folate	18	17	3	4
Vitamin B6	12	8	1	3
Methylmalonic acid	3	3	0	1
Holotranscobalamin	1	1	0	0
3-O-methyldopa	0	0	0	0

No included study reported a quantitative 3-O-methyldopa value alongside a peripheral nerve outcome, which is why the metabolite closest to the proposed mechanism contributes nothing to this review. No study reported the number of patients below a deficiency threshold separately for patients with and without peripheral neuropathy, so no odds ratio for neuropathy given deficiency could be assembled.
